# Parent-of-Origin Effects Implicate Epigenetic Regulation of Experimental Autoimmune Encephalomyelitis and Identify Imprinted *Dlk1* as a Novel Risk Gene

**DOI:** 10.1371/journal.pgen.1004265

**Published:** 2014-03-27

**Authors:** Pernilla Stridh, Sabrina Ruhrmann, Petra Bergman, Mélanie Thessén Hedreul, Sevasti Flytzani, Amennai Daniel Beyeen, Alan Gillett, Nina Krivosija, Johan Öckinger, Anne C. Ferguson-Smith, Maja Jagodic

**Affiliations:** 1 Department of Clinical Neuroscience, Center for Molecular Medicine, Karolinska Institutet, Stockholm, Sweden; 2 Department of Genetics, University of Cambridge, Cambridge, United Kingdom; University of Pennsylvania, United States of America

## Abstract

Parent-of-origin effects comprise a range of genetic and epigenetic mechanisms of inheritance. Recently, detection of such effects implicated epigenetic mechanisms in the etiology of multiple sclerosis (MS), a chronic inflammatory disease of the central nervous system. We here sought to dissect the magnitude and the type of parent-of-origin effects in the pathogenesis of experimental neuroinflammation under controlled environmental conditions. We investigated inheritance of an MS-like disease in rat, experimental autoimmune encephalomyelitis (EAE), using a backcross strategy designed to identify the parental origin of disease-predisposing alleles. A striking 37–54% of all detected disease-predisposing loci depended on parental transmission. Additionally, the Y chromosome from the susceptible strain contributed to disease susceptibility. Accounting for parent-of-origin enabled more powerful and precise identification of novel risk factors and increased the disease variance explained by the identified factors by 2-4-fold. The majority of loci displayed an imprinting–like pattern whereby a gene expressed only from the maternal or paternal copy exerts an effect. In particular, a locus on chromosome 6 comprises a well-known cluster of imprinted genes including the paternally expressed *Dlk1*, an atypical Notch ligand. Disease-predisposing alleles at the locus conferred lower *Dlk1* expression in rats and, together with data from transgenic overexpressing *Dlk1* mice, demonstrate that reduced *Dlk1* drives more severe disease and modulates adaptive immune reactions in EAE. Our findings suggest a significant epigenetic contribution to the etiology of EAE. Incorporating these effects enables more powerful and precise identification of novel risk factors with diagnostic and prognostic implications for complex disease.

## Author Summary

Even with recent progress in determining the genetic basis of complex diseases, the issue of ‘missing heritability’ remains and its potential sources are frequently speculated about but rarely explained. Parent-of-origin effects might contribute to the ‘missing heritability’ and involve genetic and epigenetic mechanisms of inheritance. Our study is the first that establishes (i) the magnitude and (ii) the type of parent-of-origin effects in the pathogenesis of a multiple sclerosis-like disease, experimental autoimmune encephalomyelitis (EAE) in rat, using a strategy designed to identify genes that confer risk only when inherited from either mother or father. A striking 37-54% of all risk loci depended on parental origin. Accounting for parent-of-origin enabled more powerful and precise identification of novel risk factors for EAE, such as the imprinted *Dlk1*gene. Disease-predisposing alleles conferred lower *Dlk1* expression in rats and transgenic *Dlk1* mice demonstrated that lower *Dlk1* drives more severe EAE and modulates adaptive immune responses. Because parental-origin effects are epigenetically regulated, our data implicate a contributory role for epigenetic mechanisms in complex diseases. Considering parent-of-origin effects in complex disease has enabled more powerful and precise identification of novel risk factors.

## Introduction

Complex diseases, like common chronic inflammatory conditions, arise from an interplay between multiple risk genes and environmental factors. Etiology is often largely unknown with variable penetrance and expressivity making it difficult to identify contributing factors.

Epigenetic mechanisms might act at the interface between the genome and environmental signals and determine stable and heritable changes in gene expression that do not require changes in the DNA sequence. Such states are mediated by DNA methylation and post-translational modifications to core histones that have an impact on gene expression [1]. Thus, it is not surprising that deregulated epigenetic mechanisms can lead to pathological conditions extensively studied in tumor biology. Therefore, while the DNA sequence confers the primary information for expression and protein structure, epigenetic mechanisms are dynamic and can mediate information about the cellular environment to regulate the specific establishment and maintenance of gene expression. Studies in mice have shown that maternal diet is associated with changes in DNA methylation in offspring [2], [3]. Additionally, monozygotic twins acquire differences in chromatin structure during their life span [4], [5]. Such altered epigenetic states might confer differences in disease susceptibility between monozygotic twins, as shown in systemic lupus erythematosus [6]. Moreover, some environmentally-associated epigenetic changes might even be transmitted through generations, as suggested in humans [7], [8] and demonstrated in mice and rats [9]–[11].

A number of recent studies implicate epigenetic mechanisms in the inheritance of multiple sclerosis (MS), a chronic inflammatory disease of the central nervous system (CNS). For example, there is a significantly higher risk for maternal half-siblings to develop MS compared to paternal half-siblings [12]. Similar parent-of-origin effects have recently been demonstrated for the major MS risk factor, the *HLA* haplotype [13], [14]. *HLA* is also under direct and indirect epigenetic regulation as DNA methylation has been demonstrated to alter the expression of *HLA* and its transcriptional activator [15]. The increasing prevalence of MS among women during the last several decades is speculated to result from changes in the environment [16] and the risk for MS is increased in children of affected mothers [17]. Thus, there is emerging evidence for complex interactions between genetic, environmental and epigenetic mechanisms underlying the pathogenesis of MS.

We here sought to dissect the extent of parent-of-origin effects in the etiology of an experimental MS-like disease, myelin oligodendrocyte glycoprotein (MOG)-induced experimental autoimmune encephalomyelitis (EAE) in rodents. We used a backcross strategy between susceptible DA and resistant PVG.AV1 rat strains designed to identify the parental origin of disease-predisposing alleles. Typically, DA rats display a relapsing-remitting disease course with an average onset around two weeks after immunization with MOG antigen, which is used to trigger the immune response in this model. Conversely, PVG.AV1 rats are nearly completely resistant to the same induction protocol [18]. Our study establishes the magnitude and the type of parent-of-origin effects in inheritance of rat EAE, a robust MS model that mimics many features of the human disease [19] providing valuable insight into the mechanism of susceptibility to complex inflammatory diseases and identifying new risk factors.

## Results

**Figure 1:**
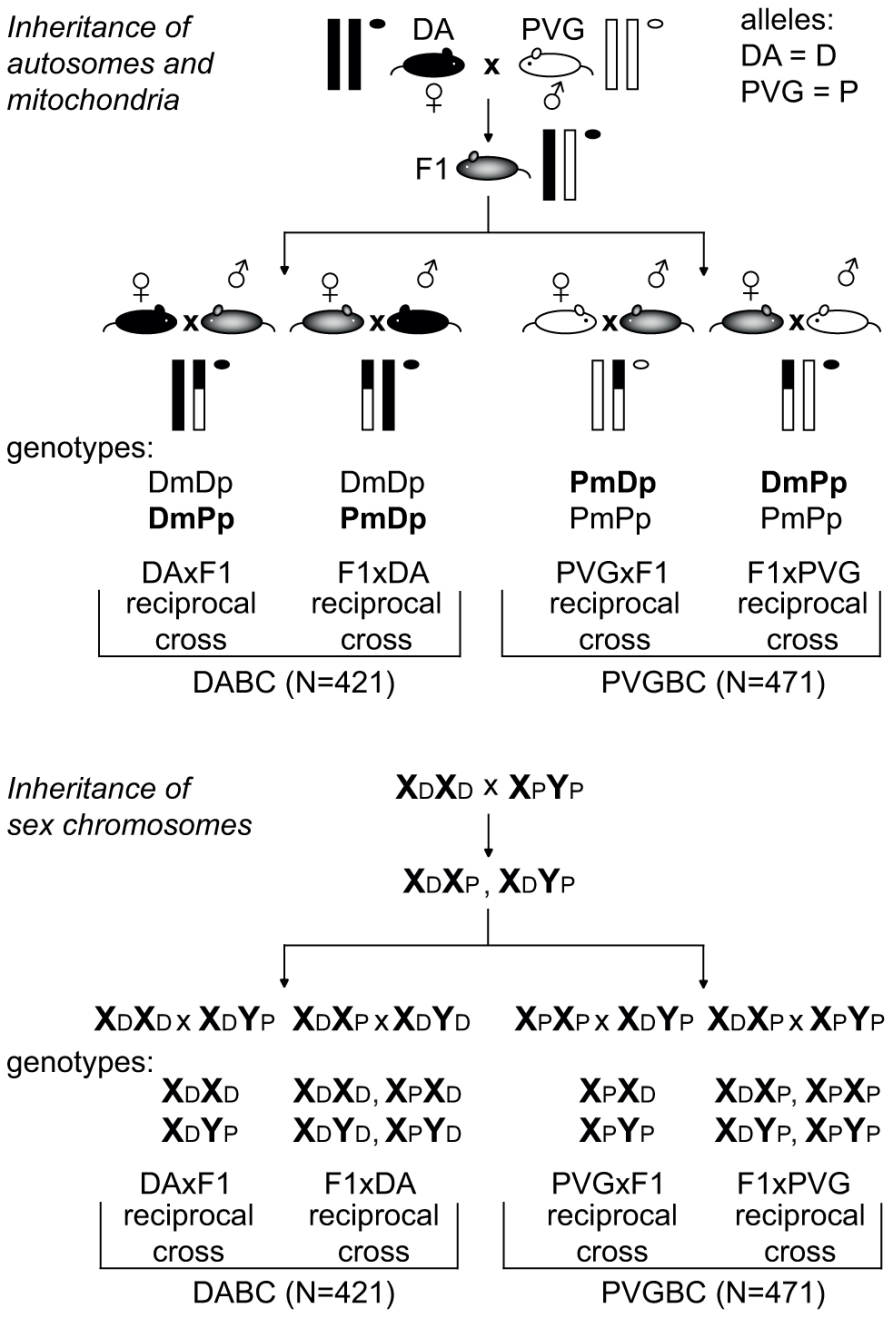
Backcross design. Schematic illustration of the experimental set-up used to breed reciprocal crosses that enabled identification of parent-or-origin effects. EAE-susceptible DA and -resistant PVG strains were backcrossed to heterozygous F1 (DAxPVG) hybrids to generate two experimental populations, DABC and PVGBC, respectively. Within each population, two reciprocal crosses were established by breeding F1 hybrids to DA mothers (DAxF1) or fathers (F1xDA) in DABC and PVG mothers (PVGxF1) or fathers (F1xPVG) in PVGBC. Transmission of autosomal chromosomes (one pair of autosomes is represented by vertical lines) and mitochondria (represented by circle) is shown in the upper panel. Transmission of sex chromosomes is shown in the lower panel. DA and PVG alleles are indicated with D and P, respectively, and the maternally and paternally inherited alleles are indicted with m and p, respectively.

Parent-of-origin effects reflect a combination of genetic and epigenetic mechanisms. In order to investigate them, we created two experimental populations by back crossing heterozygous F1 hybrids with either the susceptible DA strain (DABC, N = 421) or the resistant PVG strain (PVGBC, N = 471) (Figure 1). Within each population, two reciprocal crosses were established that were used to identify parent-of-origin dependent quantitative trait loci (QTL), while either of the two populations were used to validate QTLs identified in the other population of two reciprocal crosses.

### Genetic parent-of-origin effects: Sex chromosomes and mitochondria

Genes on the sex chromosomes are inherited in a parent-of-origin dependent manner and the influence of the Y chromosome has been well documented in mouse EAE [20], [21]. To assess the impact of the sex chromosomes in EAE in rats, we bred rats that have the X and the Y chromosome inherited either from the EAE-susceptible DA or from the EAE-resistant PVG rat strain (Figure 1). The F1xDA male offspring (N = 104) that carry the susceptible DA Y chromosome had an overall higher EAE incidence with earlier disease onset and more severe disease than DAxF1 offspring males (N = 104) that carry resistant PVG Y chromosome (Table 1). Thus, the Y chromosome from the susceptible DA strain conferred higher incidence and more severe EAE. This is further supported by absence of a difference in disease susceptibility between PVGxF1 (N = 106) and F1xPVG (N = 126) offspring males that inherited the Y chromosome from the resistant PVG strain (Table 1, Figure 1). Potential interactions between Y and nuclear genes could not be explored due to the backcross design where the Y effect in the DABC population segregates between reciprocal crosses together with the parent-of-origin effects. Furthermore, we did not observe any global influence of the X chromosome in this study (Table 1).

**Table 1:** Comparisons of reciprocal crosses within DABC and PVGBC populations.

	DABC Females			DABC Males		
	DAxF1 N = 105	F1xDA N = 108	p-value	DAxF1 N = 104	F1xDA N = 104	p-value
**Weight**	105.7 (12.7)	108.9 (11.8)	ns	167.3 (24.3)	180.4 (19.8)	3×10^−5^
**INC^a^**	0.61 (0.5)	0.67 (0.49)	ns	0.60 (0.51)	0.77 (0.47)	0.02
**MAX^b^**	1.98 (1.65)	1.91 (1.52)	ns	1.68 (1.53)	2.21 (1.43)	0.01
**DUR^b^**	13.17 (11.88)	12.39 (10.96)	ns	11.7 (11.56)	16.18 (11.71)	0.006
**ONS^b^**	21.25 (11.97)	20.72 (12.21)	ns	22.42 (12.95)	19.04 (13.13)	ns
**WL^b^**	6.64 (12.33)	4.53 (10.94)	ns	5.05 (11.56)	10.01 (13.49)	0.005

	PVGBC Females			PVGBC Males		
	PVGxF1 N = 119	F1xPVG N = 120	p-value	PVGxF1 N = 106	F1xPVG N = 126	p-value
**Weight**	112.1 (14)	108.7 (13.3)	ns	204.3 (28.3)	202 (25.4)	ns
**INC^a^**	0.68 (0.50)	0.62 (0.52)	ns	0.61 (0.51)	0.65 (0.53)	ns
**MAX^b^**	2.2 (1.8)	2.1 (1.9)	ns	2.2 (1.9)	2.3 (1.9)	ns
**DUR^b^**	6.7 (5.5)	6.3 (5.6)	ns	5.5 (5.1)	6.3 (5.4)	ns
**ONS^b^**	16.1 (6.2)	16.6 (6.3)	ns	17.4 (6.2)	16.7 (6)	ns
**WL^b^**	5.5 (33.5)	8.6 (53.6)	ns	4.7 (47.4)	9.1 (57.2)	ns

Weight and clinical EAE phenotypes were compared between reciprocal backcrosses to assess global differences. In DABC, F1xDA males (DA Y chromosome) were significantly different from DAxF1 males (PVG Y chromosome), while there were no differences between other reciprocal pairs. The mean value is given with standard deviation in parenthesis. ^a^Fisher's Exact Test, ^b^Student's t-test, p-values ≤0.05 were considered significant. Abbreviations: Weight  =  weight in grams on the day of immunization, INC  =  incidence of EAE, MAX  =  maximum EAE score, DUR  =  duration of EAE, ONS  =  onset of EAE, WL  =  weight loss, ns  =  not significant.

Exclusively maternally inherited are genes encoded by the mitochondrial genome. To date, the influence of mitochondria has not been unequivocally established in EAE [22]. To assess the impact of mitochondria on EAE in rats, we bred rats that inherited mitochondria either from the DA or from the PVG strain (Figure 1). We did not detect robust differences between rats with varying mitochondrial genomes (Table 1, Text S1).

Therefore considering parental effects of sex chromosomes and mitochondria, the Y chromosome is primarily responsible for MOG-induced EAE in these rat strains.

### Parent-of-origin effects at autosomes: Imprinted loci

In the next stage, we investigated the impact of epigenetic mechanisms such as imprinting on inheritance of EAE. To that end, we identified genome-wide QTLs, which are genomic loci that encode EAE risk genes with the disease status dependent on the genotype at these loci (presence of either DA = D or PVG = P alleles). To identify parent-of-origin dependent QTLs, we used a reciprocal backcross breeding strategy that allowed the risk alleles inherited from mothers to be unequivocally discriminated from those of fathers in the heterozygous animals [23]. QTLs detected in DABC were considered as parent-of-origin dependent QTLs if they showed an effect in only one of the reciprocal crosses, *either* DAxF1 (PVG alleles are paternally inherited) *or* F1xDA (PVG alleles are maternally inherited). We also detected overlapping or additional parent-of-origin QTLs in PVGBC that showed an effect in only one of the reciprocal crosses, *either* PVGxF1 (DA alleles are paternally inherited) *or* F1xPVG (DA alleles are maternally inherited). This pattern of inheritance, whereby a gene variant affects the phenotype only when expressed from the maternal or the paternal copy is typical for imprinted genes.

### Proof of concept for reciprocal backcross strategy: A parent-of-origin dependent QTL comprises the imprinted *IGF2* gene

The reciprocal backcross strategy was first tested on the weight of naïve rats, which is a physiological phenotype not related to EAE. PVG rats are typically heavier than age-matched DA rats, and several QTLs regulate this trait in DABC (Figure S1A). We detected a QTL on chromosome 1 in females with the peak around 185 Mb (Figure S1B) where the PVG allele predisposed for higher weight preferentially when paternally inherited (Figure S1C). The same locus predisposed for higher weight when paternally inherited in the validation population of PVGBC females (p<0.001).

The *IGF2* gene, known to regulate growth and weight and to be expressed only from the paternal copy [24], is encoded in this locus. Concordantly, the weight of newborn F1 hybrid pups was higher when the PVG allele was paternally inherited (p<0.05) (Figure S1D). To confirm *IGF2* imprinting in our rat strains, we measured allele-specific *IGF2* expression utilizing a single nucleotide polymorphism (SNP) in the 5′ UTR of *IGF2* that segregates between DA and PVG (Figure S1E). *IGF2* was exclusively expressed from the paternal copy in livers of newborn rats. Together, these data suggest that the gene responsible for weight regulation in a parent-of-origin manner in this QTL is likely *IGF2*. It also shows that the reciprocal backcross strategy can be used to identify parent-of-origin dependent QTLs.

### Identification and validation of parent-of-origin dependent QTLs in EAE

We then used the reciprocal backcross strategy to carry out identification of QTLs that control EAE susceptibility (reflected by incidence and onset), EAE severity (reflected by maximum score and duration) and subclinical disease (reflected by weight loss). We performed genome-wide linkage analysis in DABC and PVGBC populations using forward selection followed by backward elimination model in R/qtl [25]). In total, 16 and 11 loci showed evidence for linkage with different EAE phenotypes in DABC and PVGBC, respectively (Table S1). We next performed genome-wide linkage analysis in the separate reciprocal crosses to identify parent-of-origin dependent QTLs. All reciprocal crosses had similar and sufficient power to detect QTLs of effects typical for EAE (Table S2, Table S3). Of all EAE QTLs, 44% (7/16) and 73% (8/11) showed parent-of-origin dependent transmission of risk alleles in DABC and PVGBC, respectively (Table 2, Table 3). The experimental design, comprising two independent populations (DABC and PVGBC), allows validation of identified parent-of-origin dependent QTLs (Figure 1). Six out of 8 (75%) QTLs that showed linkage in only one of the reciprocal crosses in the DABC population (Table 2) also showed evidence for linkage in one of the reciprocal crosses within the independent PVGBC population (Table 3), with the same type of parental transmission of risk, i.e. maternal or paternal. Likewise, six out of 8 (75%) QTLs, identified in only one reciprocal cross in the PVGBC population (Table 3) were confirmed in one of the reciprocal crosses within the independent DABC population (Table 2), with the same type of parental transmission.

**Table 2:** Allelic effects and transmission of QTLs mapped in the DABC population.

	DABC			DA x F1			F1 x DA			Allele	Cross	Trans
QTL	ONS^a^	MAX^b^	WL	ONS^a^	MAX^b^	WL	ONS^a^	MAX^b^	WL			
**Females**	N = 213			N = 105			N = 108					
1b:248	ns	ns	ns	ns	ns	ns	ns	ns	0.04	P	ALL	
4a:144*	0.002	0.03	0.01	ns	ns	ns	**9×10−5**	**0.001**	**0.008**	D	F1xDA	Mat
4b:185	0.001	0.002	0.006	0.02	ns	ns	0.02	ns	0.02	D	ALL	
5b:157	0.02	0.004	0.003	ns	ns	ns	**0.001**	**0.0001**	**0.0001**	P	F1xDA	Mat
6:131*	ns	ns	0.02	**0.03**	ns	**0.01**	ns	ns	ns	P	DAxF1	Pat
7a:21	0.03	0.03	ns	ns	0.04	0.05	ns	ns	ns	P	ALL	
7b:50*	ns	ns	ns	ns	ns	ns	ns	**0.03**	**0.03**	D	F1xDA	
10d:98	0.02	0.005	ns	ns	0.02	ns	0.05	ns	ns	D	ALL	
12:25	0.003	0.0004	5×10−5	0.01	0.004	0.0006	ns	0.04	0.02	D	ALL	
14:5	0.02	ns	ns	ns	ns	ns	**0.0003**	**0.02**	ns	P	F1xDA	Mat
**Males**	N = 208			N = 104			N = 104					
1b:248	ns	0.002	8×10−5	ns	ns	0.04	ns	0.01	0.007	P	ALL	
3:161*	ns	ns	ns	ns	ns	ns	ns	**0.04**	**0.05**	D	F1xDA	Mat
4a:144*	ns	ns	ns	ns	ns	ns	ns	ns	**0.006**	D	F1xDA	Mat
10a:23*	0.004	0.006	ns	ns	0.03	ns	0.03	ns	ns	D	ALL	Mito?
10b:50*	0.004	0.007	0.01	ns	ns	ns	0.008	ns	ns	D	ALL	
11:47	0.008	0.04	0.004	0.01	0.05	0.007	ns	ns	ns	D	ALL	
18:80*	ns	ns	ns	ns	ns	ns	**0.02**	ns	ns	P	F1xDA	Mat
19:50	0.009	0.01	0.02	ns	ns	ns	ns	ns	ns	D	ALL	

QTLs are indicated with chromosome: location in Mb, *indicates QTLs that are shared between DABC and PVGBC populations. The p-values from effect plots in DABC are given, analyzed by Student's t-test, p-values ≤0.05 were considered significant. ^a^ Onset and incidence gave similar results and ONS is shown as a representative for susceptibility phenotypes. ^b^ Maximum and cumulative EAE scores and duration of EAE showed similar results and MAX represents the severity phenotypes. Bold indicates significant p-values for QTLs showing evidence for parent-of-origin dependence. Abbreviations: ONS  =  onset of EAE, MAX  =  maximum EAE score, WL  =  weight loss, Allele  =  disease predisposing allele, D  =  DA, P  =  PVG, Trans  =  transmission of disease-predisposing allele, Cross  =  indicates reciprocal cross in which QTL was identified, Mat  =  maternal transmission, Pat  =  paternal transmission and Mito  =  mitochondria.

**Table 3:** Allelic effects and transmission of QTLs mapped in the PVGBC population.

	PVGBC			PVG x F1			F1 x PVG			Allele	Cross	Trans
QTL	ONS^a^	MAX^b^	WL	ONS^a^	MAX^b^	WL	ONS^a^	MAX^b^	WL			
**Females**	N = 239			N = 119			N = 120					
3:161*	0.01	0.01	ns	ns	ns	ns	**0.003**	**0.001**	**0.05**	D	F1xPVG	Mat
4a:144*	0.04	ns	ns	ns	ns	ns	**0.003**	**0.01**	ns	D	F1xPVG	Mat
6:131*	ns	0.05	ns	**0.05**	ns	ns	ns	ns	ns	P	PVGxF1	Pat
7b:50*	ns	ns	ns	**0.04**	**0.02**	ns	ns	ns	ns	D	PVGxF1	
10a:23*	0.007	0.03	ns	ns	ns	ns	**0.003**	**0.01**	ns	D	F1xPVG	Mito?
10b:50*	0.005	0.009	0.01	ns	ns	ns	0.04	0.008	0.002	D	ALL	
10c:82	ns	ns	0.02	**0.003**	**0.009**	**0.04**	ns	ns	ns	D	PVGxF1	Pat
**Males**	N = 232			N = 106			N = 126					
1a:25	ns	ns	0.03	ns	ns	ns	**0.02**	**0.006**	**0.002**	P	F1xPVG	Mat
4a:144*	0.002	0.02	0.02	ns	ns	ns	**0.0003**	**0.001**	**0.04**	D	F1xPVG	Mat
6:131*	0.04	ns	ns	**0.002**	**0.006**	ns	ns	ns	ns	P	PVGxF1	Pat
10a:23*	ns	ns	ns	ns	ns	ns	0.04	ns	ns	D	F1xPVG	Mito?
10b:50*	0.005	ns	ns	ns	ns	ns	0.005	0.02	ns	D	ALL	
10c:82	ns	ns	ns	ns	**0.05**	ns	ns	ns	ns	D	PVGxF1	Pat
18:80*	ns	ns	ns	ns	ns	ns	**0.004**	**0.003**	**0.002**	P	F1xPVG	Mat

QTLs are indicated with chromosome: location in Mb, * indicates QTLs that are shared between DABC and PVGBC populations. The p-values from effect plots in PVGBC are given, analyzed by Student's t-test, p-values ≤0.05 were considered significant. ^a^ Onset and incidence gave similar results and ONS is shown as a representative for susceptibility phenotypes. ^b^ Maximum and cumulative EAE scores and duration of EAE showed similar results and MAX represents the severity phenotypes. QTLs on chromosome 5a (25 Mb) and 13 (58 Mb) (Table S1) were significant only in the entire PVGBC and were therefore taken out of the table. Bold indicates significant p-values for QTLs showing evidence for parent-of-origin dependence. Abbreviations: ONS  =  onset of EAE, MAX  =  maximum EAE score, WL  =  weight loss, Allele  =  disease predisposing allele, D  =  DA, P  =  PVG, Trans  =  transmission of disease-predisposing allele, Cross  =  indicates reciprocal cross in which QTL was identified, Mat  =  maternal transmission, Pat  =  paternal transmission and Mito  =  mitochondria.

To additionally confirm that these are true parent-of-origin QTLs and not a failure to detect a significant QTL in one of the crosses, we performed analysis of cross-by-QTL interactions, which is a statistical way of showing that the parental origin of the allele (inferred by the “cross”) affects the expressivity of the QTL. The majority of identified parent-of-origin QTLs (80%) showed significant dependence on the cross/origin (Table 4). Only a QTL on chromosome 10 (peak at 23 Mb) failed to show interaction and a QTL on chromosome 7 (peak at 50 Mb) showed significance only for one phenotype.

**Table 4:** Cross-by-QTL interaction analysis in DABC and PVGBC.

Pheno	1a:25^a^	3:161^b(c)^	4a:144^d(a,b)^	5b:157^d^	6:131^a(d)^	7b:50^d^	10a:23^b^	10c:82^b^	14:5^d^	18:80^a^
INC	**0.036**	**0.031**	**0.031**	n/a	**0.019**	n/a	0.487	n/a	**0.035**	**0.037**
MAX	**0.033**	**0.028**	**0.036**	**0.025**	**0.034**	n/a	0.486	n/a	n/a	**0.039**
DUR	n/a	n/a	n/a	**0.029**	**0.006**	n/a	0.176	**0.031**	n/a	**0.021**
ONS	n/a	0.101	**0.048**	0.093	**0.018**	0.686	0.181	**0.009**	**0.020**	n/a
WL	**0.020**	n/a	0.260	**0.047**	n/a	**0.027**	n/a	n/a	n/a	**0.040**

The statistical validation of the cross-by-QTL interaction was performed using the fit-multiple QTL analysis (for details see Materials and Methods). A full model comprised all identified QTLs (from forward selection with reverse elimination, as in Table S1) and CROSS x QTL interaction terms for the QTLs that displayed parent-of-origin effect (i.e. QTLs that could be mapped only in one of the crosses). In the next stage the effect of each QTL or CROSS x QTL interaction was subtracted from the full model and the contribution of the subtracted term to the full model was evaluated and expressed in p-values. The model used was Phenotype ∼ QTL1a + QTL1a*CROSS + QTL1b + QTL3 + QTL3*CROSS + QTL4a + QTL4a*CROSS + QTL4b + QTL5b + QTL5b*CROSS + QTL6 + QTL6*CROSS + QTL7a + QTL7b + QTL7b*CROSS + QTL10a + QTL10a*CROSS + QTL10b + QTL10c + QTL10c*CROSS + QTL10d + QTL11 + QTL12 + QTL14 + QTL14*CROSS + QTL18 + QTL18*CROSS + QTL19 + ε. Presented in the table are only p-values for the parent-of-origin dependent QTLs x CROSS terms. The p-values from the fit-multiple QTL analysis in the PVGBC males^a^ (N = 232), PVGBC females^b^ (N = 239), DABC males^c^ (N = 208) and DABC females^d^ (N = 213) are given. The population with the most significant p-values is shown for each QTL and specified in superscript, with the additional populations that show significant CROSS x QTL interaction indicated in superscript parenthesis. n/a, analysis could not be applied due to lack of evidence of a QTL.

Nonetheless, we wanted to confirm the parent-of-origin dependent QTLs in an entirely different experimental population. For that purpose we used 794 rats originating from the same DA and PVG.AV1 parental strains that were randomly bred for 10 generations (G10) and induced with EAE. We repeated the QTL interaction analysis described above in this large population. We found a significant origin-by-QTL interaction in G10 for the same QTLs that showed evidence of cross-by-QTL interaction in the backcrosses (Table 5). Only two out of 10 QTLs, on chromosomes 6 and 18, could not be tested because there was no significant evidence for a QTL in G10. To confirm that parent-of-origin QTLs were not just randomly detected effects, we tested loci (N = 9) that represent main-effect EAE QTLs that did not show evidence of parent-of-origin in the backcross (Table S4) and randomly selected loci (N = 10) (Table S5) for origin-by-QTL interaction. None of the main-effect QTLs in G10 or randomly selected loci showed a level of evidence that was considered to be substantial enough for parent-of-origin QTLs. These analyses demonstrate that the majority of detected parent-of-origin QTLs is genuine. Considering only QTLs that demonstrated significant interaction with the cross/origin, 37% (6/16) and 54% (6/11) of loci displayed significant parent-of-origin dependent transmission of risk alleles in DABC and PVGBC populations, respectively (summarized in Table 6).

**Table 5:** Origin-by-QTL interaction analysis in the (DAxPVG) G10 population.

Pheno	1a:25^a^	3:161^a^	4a:144	5b:157	6:131	7b:50	10a:23	10c:82	14:5^b^	18:80
INC	**0.006**	**0.03**	**0.0003**	**0.003**	n/a	0.2	0.3	**0.005**	**0.0006**	n/a
MAX	**0.05**	**0.004**	**0.0009**	**0.005**	n/a	n/a	0.5	**0.006**	**0.003**	n/a
DUR	0.1	**0.005**	**0.002**	**0.003**	n/a	n/a	0.5	**0.005**	**0.02**	n/a
ONS	**0.04**	**0.03**	**0.00001**	**0.0007**	n/a	**0.04**	0.5	**0.0006**	**0.02**	n/a
WL	**0.01**	**0.03**	**0.03**	**0.005**	n/a	n/a	n/a	**0.04**	**0.01**	n/a

The statistical confirmation of the parent-of-origin dependent QTLs identified in the backcross populations. Analysis was performed using the fit-multiple QTL model. A full model comprised all QTLs and Parent-of-origin (G9) x QTL interactions (Phenotype ∼ QTL1a + QTL1a*ORIGIN + QTL3 + QTL3*ORIGIN + QTL4a + QTL4a*ORIGIN + QTL5b + QTL5b*ORIGIN + QTL7b + QTL7b*ORIGIN + QTL10a + QTL10a*ORIGIN + QTL10c + QTL10c*ORIGIN + QTL14 + QTL14*ORIGIN + ε). In the next stage the effect of each QTL or origin x QTL interaction was subtracted from the full model and the contribution of the subtracted term to the full model was evaluated and expressed in p-values. Analysis was performed in 794 G10 rats comprising 366 males**^a^** and 428 females**^b^**. n/a, analysis could not be applied due to lack of evidence for a QTL.

**Table 6:** Summary of the parent-of-origin effects detected in QTLs.

		Total	% PoO	% Maternal
**DABC**	Females	8	37 (3/8)	67 (2/3)
	Males	6	33 (2/6)	100 (2/2)
	Females and Males	2	50 (1/2)	100 (1/1)
	Total	16	37 (6/16)	83 (5/6)
**PVGBC**	Females	2	50 (1/2)	100 (1/1)
	Males	2	100 (2/2)	100 (2/2)
	Females and Males	7	43 (3/7)	33 (1/3)
	Total	11	54 (6/11)	67 (4/6)
**Shared DABC and PVGBC**		**7**	**57 (4/7)**	**75 (3/4)**

Abbreviations: % PoO  =  *percent* of QTLs that show parent-of-origin effect (numbers are given in parenthesis), % Maternal  =  percent of parent-of-origin effect QTLs that show maternal transmission of the disease-predisposing allele (numbers are given in parenthesis).

**Figure 2:**
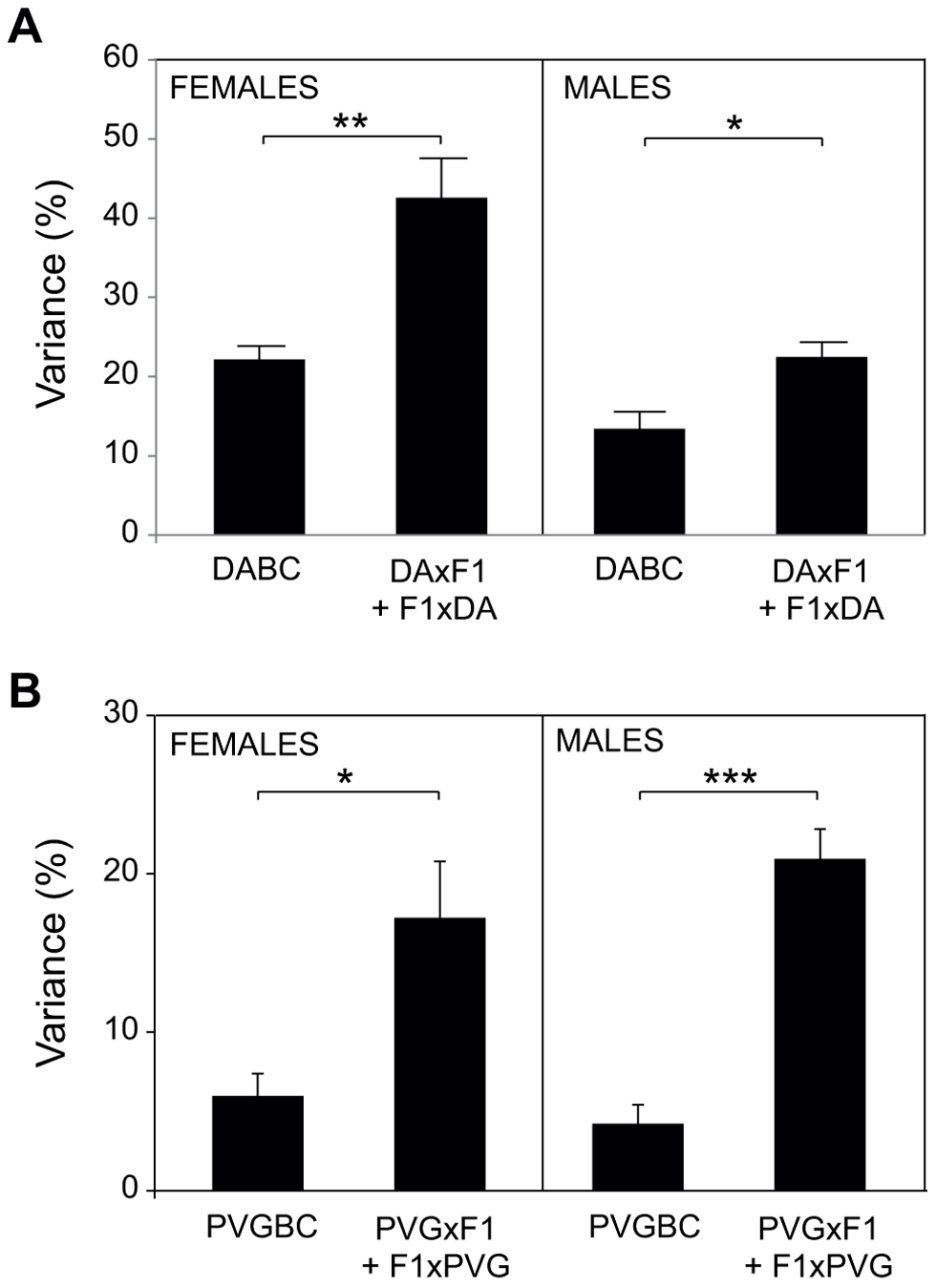
Accounting for parent-of-origin effects improves disease inheritance models. Phenotypic variance (%) that can be explained by risk loci is greatly increased when parent-of-origin effects are included, supporting their contribution to EAE. The fit multiple-QTL model was used to validate the independent effects of each QTL (phenotype  =  *QTL1 + QTL2 + … + QTLn +ε*) (Table S1, Table S6). A) Mean variance of all phenotypes +/− SEM is presented for the entire DABC and as a sum of variance for each reciprocal cross (DAxF1) + (F1xDA). Due to the high severity in DABC males, the severity phenotypes are omitted. B) Mean variance of all phenotypes +/− SEM is presented for the entire PVGBC and as a sum of variance for each reciprocal cross (PVGxF1) + (F1xPVG).*, ** and *** indicate p<0.05, 0.01 and 0,001, respectively.

Moreover, accounting for the parent-of-origin defines risk factors that explain a 2–4 fold higher percentage of disease variance compared to the factors identified in populations where parental origin is not considered (Figure 2, Table S1, Table S6). Contribution from more QTLs can be established if analyses are done in separate reciprocal crosses (Table S1).

### Maternal transmission of EAE-predisposing alleles

**Figure 3:**
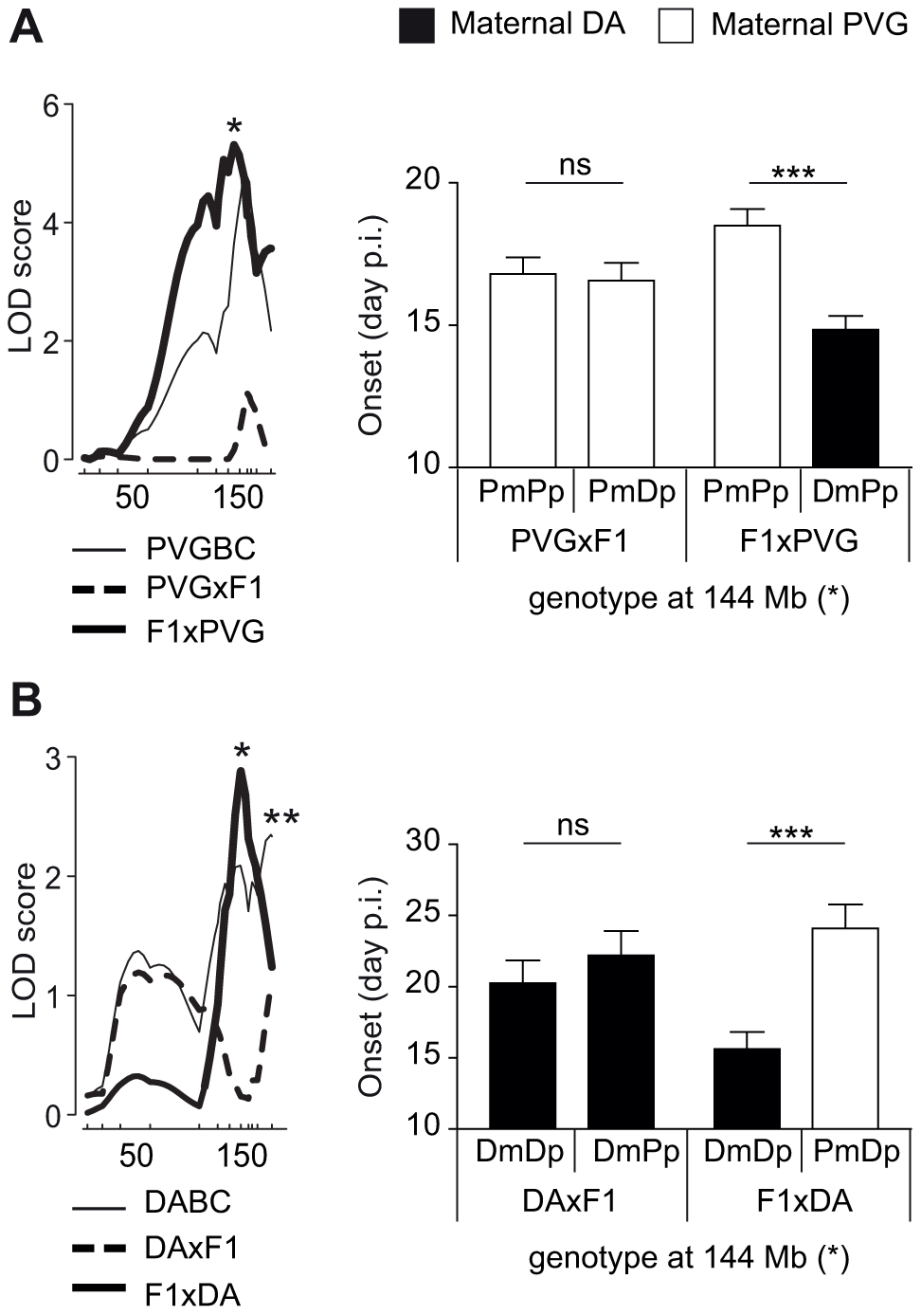
Maternal transmission of the disease-predisposing allele. A) A logarithm of the odds (LOD) plot of chromosome 4 shows linkage for disease onset in F1xPVG offspring (peak at 144 Mb indicated by *) but not in PVGxF1 offspring, with the maternally transmitted DA allele predisposing for earlier onset of disease compared to the PVG allele. B) The same QTL at 144 Mb regulated onset of disease in DABC in F1xDA offspring but not in DAxF1 offspring, showing maternal transmission of the disease-predisposing DA allele in the independent DABC population (peak at 144 Mb indicated by *). An additional QTL that did not show parent-of-origin dependent effect was identified in DABC population (peak at 185 Mb indicated by **). Onset of EAE is shown as representative of all clinical phenotypes. LOD scores were generated using Haley-Knott regression (genome-wide p<0.05 thresholds for significant linkage were 2.6, 2.6, 2.8, 2.9, 2.8 and 2.8 for PVGBC (N = 471), PVGxF1 (N = 225), F1xPVG (N = 246), DABC females (N = 213), DAxF1 females (N = 105) and F1xDA females (N = 108), respectively). P-values given in allelic effect plots were calculated using Student's t-test (p-value <0.001  =  ***, ns  =  not significant). DA and PVG alleles are indicated with D and P, respectively, and the maternally and paternally inherited alleles are indicted with m and p, respectively.

We identified parent-of-origin QTLs with maternal transmission of EAE-predisposing alleles only in the F1xDA and F1xPVG reciprocal crosses, i.e. QTLs on chromosomes 1, 3, 4, 5, 14 and 18 (Table 2, Table 3). A QTL on chromosome 4 (peak at 144 Mb) was identified in two independent experimental populations (DABC and PVGBC) with the DA allele predisposing for disease only when maternally inherited. In PVGBC, the DA allele predisposed for EAE only in F1xPVG offspring, which inherited the DA allele maternally (Figure 3A). Interestingly, in DABC females, analysis in reciprocal backcrosses separated what originally appeared to be one wide QTL, with a peak at 185 Mb, into two QTLs at 144 Mb and 185 Mb (Table 2, Figure 3b). The first of them overlapped the QTL identified in PVGBC (peak at 144 Mb) and also displayed linkage in only one of the reciprocal crosses, F1xDA. Additionally, this QTL displayed significant cross-by-QTL interactions in all three populations, DABC, PVGBC and G10 for multiple phenotypes (Table 4, Table 5). The genetic variants (EAE-promoting DA vs. EAE-protective PVG) at this QTL could exert the effect on EAE only when present on the maternally inherited chromosome, resembling genomic imprinting. This pattern of transmission of risk alleles implicates that the underlying gene is preferentially expressed from the maternal copy while it is fully or partially repressed on the paternal copy.

### Paternal transmission of EAE-predisposing alleles

**Figure 4:**
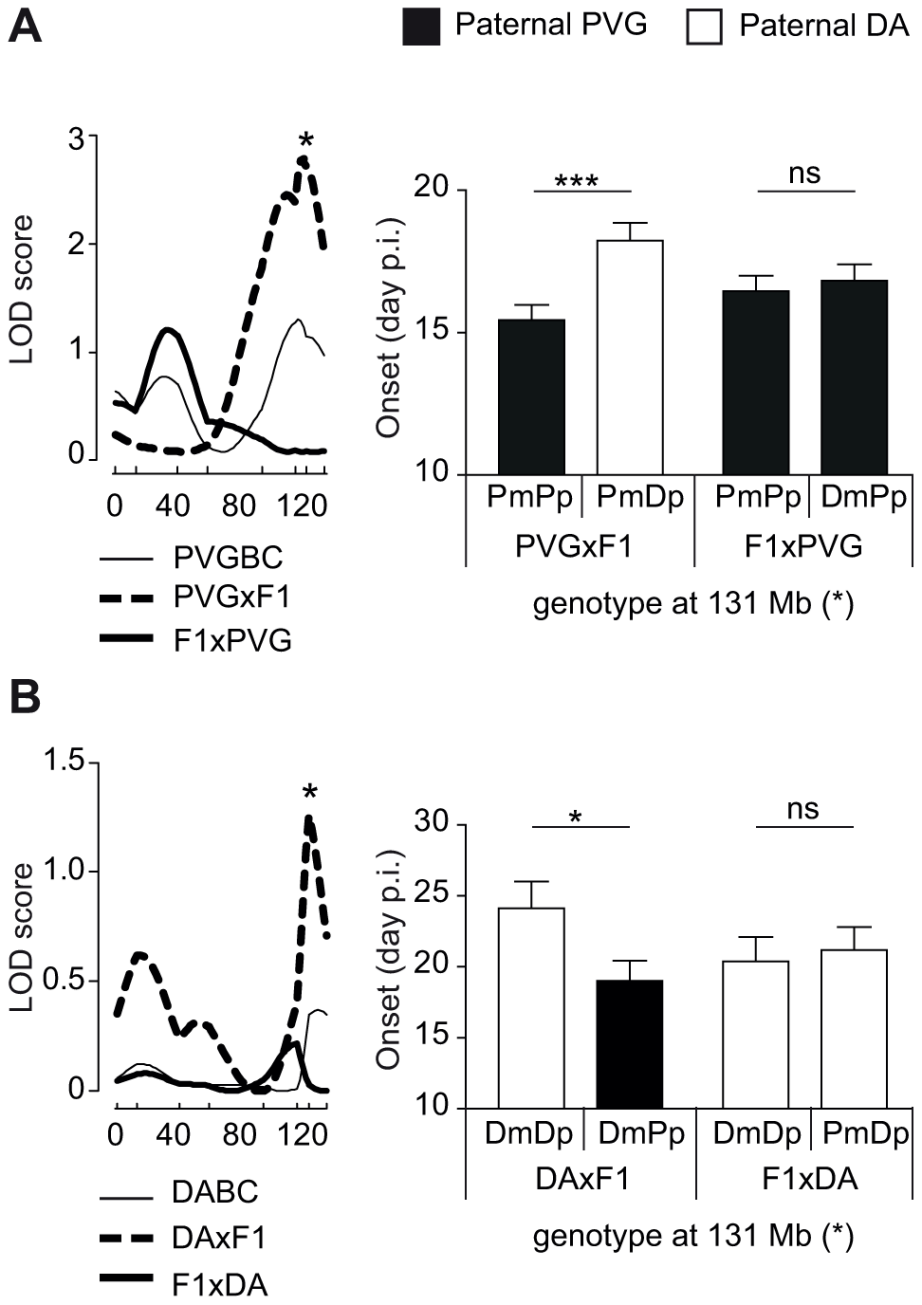
Paternal transmission of the disease-predisposing allele. A) A logarithm of the odds (LOD) plot of chromosome 6 shows linkage for disease onset in PVGxF1 offspring (peak at 131 Mb indicated by *) but not in all PVGBC or F1xPVG offspring, with the paternally transmitted PVG allele predisposing for earlier onset of disease compared to the DA allele. B) The same QTL at 131 Mb displayed evidence for linkage in DABC females in DAxF1 offspring but not in F1xDA offspring, showing paternal transmission of the disease-predisposing PVG allele in the independent DABC population. Onset of EAE is shown as representative of all clinical phenotypes. LOD scores were generated using Haley-Knott regression (genome-wide p<0.05 thresholds for significant linkage were 2.7, 2.8 and 2.7 for combined PVGBC (N = 471), PVGxF1 (N = 225) and F1xPVG (N = 246), respectively; nominal p<0.05 threshold for evidence of linkage was 1.3 in DAxF1 (N = 105)). P-values given in allelic effect plots were calculated using Student's t-test (p-value <0.05  =  *, p-value <0.001  =  ***, ns  =  not significant). DA and PVG alleles are indicated with D and P, respectively, and the maternally and paternally inherited alleles are indicted with m and p, respectively.

We then investigated parent-of-origin QTLs that depend on paternal transmission, identified only in the DAxF1 and PVGxF1 reciprocal crosses, i.e. QTLs on chromosomes 6, 7 and 10 (Table 2, Table 3). A QTL on chromosome 6 (peak at 131 Mb) was identified in PVGBC and confirmed in DABC females (Table 3, Table 2). In PVGBC, the paternal PVG allele predisposed for EAE only in PVGxF1 offspring (Figure 4A). Accordingly, in DABC, the PVG allele predisposed for EAE only in DAxF1 offspring, which inherited the PVG allele paternally (Figure 4B). The QTL displayed significant cross-by-QTL interaction in the backcross population (Table 4). The genetic variants (DA vs. PVG) at this QTL could exert the effect on EAE only when present on the paternally inherited chromosome, resembling genomic imprinting. This pattern of transmission of risk alleles implicates that the underlying gene is preferentially expressed from the paternal copy while it is fully or partially repressed on the maternally inherited copy.

### *Dlk1* is a candidate gene underlying paternally transmitted EAE QTL on rat chromosome 6

**Figure 5:**
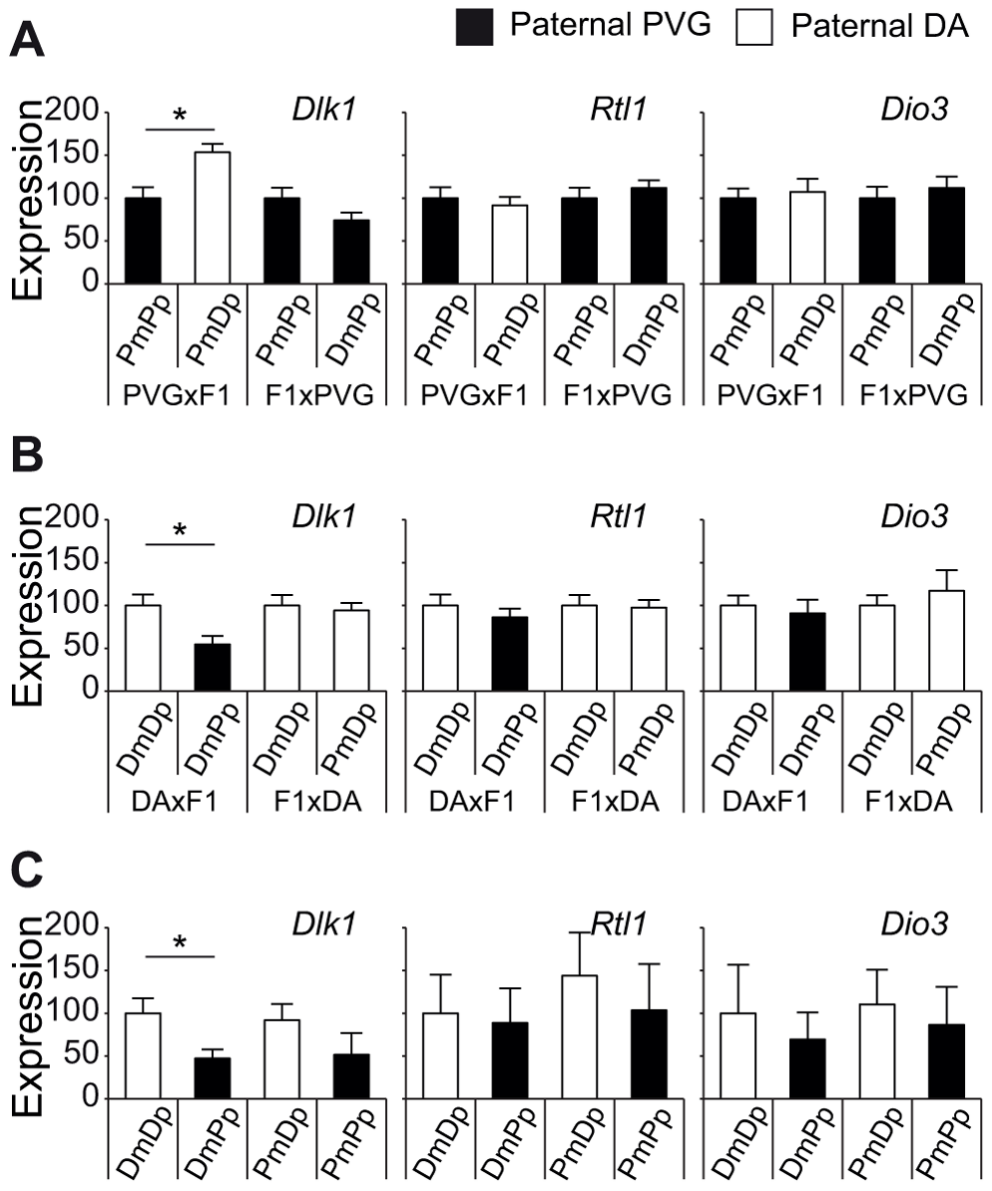
Paternally transmitted PVG allele at the Dlk1 locus predisposes for lower Dlk1 expression. *Dlk1*, *Rtl1* and *Dio3* mRNA expression in spleen tissue collected A) 21 days after EAE induction in PVGBC population (N = 372), B) 35 days after EAE induction in DABC population (N = 347), and in C) three weeks old naïve DA, PVG and reciprocal F1 hybrid rats (N = 8–9 rats/group). A and B) Rats stratified according to the genotype at the peak of the QTL on chromosome 6 (131 Mb) showed that the paternally inherited PVG allele predisposes for lower expression of *Dlk1* compared to the DA allele, whereas no effect was observed on *Rtl1* and *Dio3* expression. C) Likewise, F1 hybrids with paternally inherited PVG alleles demonstrated lower *Dlk1* expression compared to DA alleles, whereas no effect was observed on *Rtl1* and *Dio3* expression. Relative *Dlk1* expression was calculated in relation to the mean of housekeeping gene, hypoxanthine phosphoribosyltransferase (*Hprt*), using 2-ΔΔCt method and normalized within each group. P-values were calculated using Student's t-test (p-value <0.05  =  *). DA and PVG alleles are indicated with D and P, respectively, and the maternally and paternally inherited alleles are indicted with m and p, respectively.

QTL confidence intervals in backcross populations usually comprise large genomic intervals. To narrow the QTL on chromosome 6, we used probability mapping. The most likely interval to harbor the EAE-predisposing gene was between 130–134 Mb and 131–134 Mb in PVGBC and DABC, respectively. This region overlaps with a well-known cluster of imprinted genes, *Dlk1-Dio3*, on rat chromosome 6 and syntenic mouse and human chromosomes 12 and 14, respectively [26]–[28]. The predicted intergenic differentially methylated region (IG-DMR), known to control the imprinting status of the locus [29], showed around 50% methylation in spleens of backcross rats, which is typical for imprinted genes (data not shown). Thus, paternally expressed genes in the cluster, *Dlk1*, *Rtl1* and *Dio3* [28], [30], could explain paternal transmission of EAE risk allele at chromosome 6. We did not find any coding SNPs between DA and PVG in the *Dlk1*, *Rtl1* and *Dio3* genes (whole-genome SOLiD sequencing, Bäckdahl *et al*, manuscript). Therefore, we investigated if their expression levels are under parent-of-origin dependent regulation. Indeed, the PVG risk allele predisposed for lower levels of *Dlk1* in spleen compared to DA alleles only when paternally transmitted (Figure 5). Rats that inherited the PVG allele from their fathers had lower expression of *Dlk1* compared to rats with paternally inherited DA allele, in the two independent DABC and PVGBC populations (Figure 5A, B). This was further confirmed in reciprocal F1 hybrids with offspring rats that inherited PVG allele from their father displaying lower *Dlk1* expression in spleen compared to rats with paternally inherited DA allele (Figure 5C). We found no evidence for parent-of-origin dependent expression differences of *Rtl1* and *Dio3* (Figure 5). Additionally, *Dlk1* has previously been shown to be involved in regulation of immune responses [31]–[33]. Taken together, these findings suggest that *Dlk1* may at least in part, be responsible for the effect of the parent-of-origin dependent QTL on chromosome 6, and that PVG alleles can promote EAE by means of lower *Dlk1* expression when paternally inherited.

### *Dlk1* controls EAE and immune responses in mice

**Figure 6:**
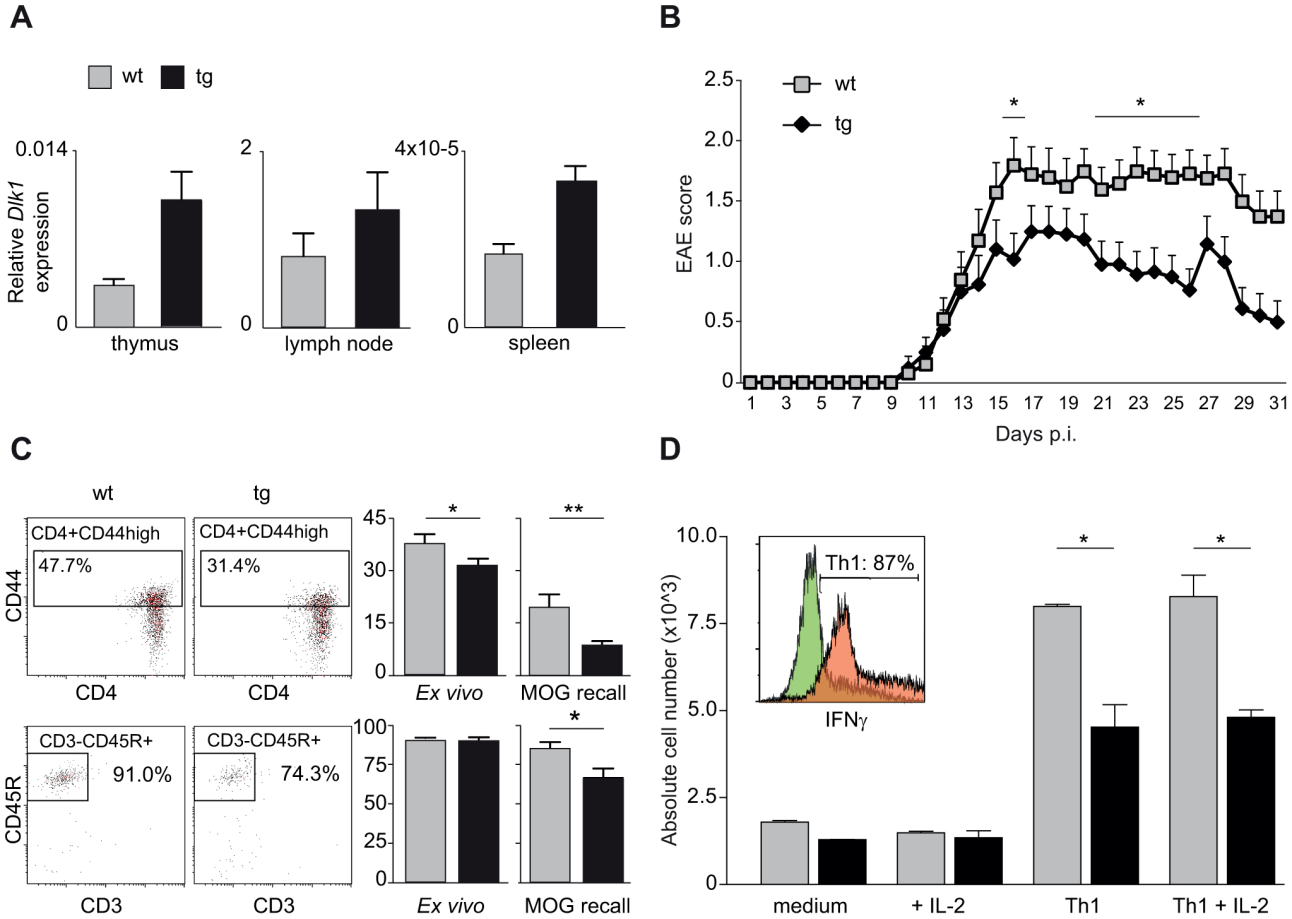
Transgenic overexpression of Dlk1 modulates EAE severity and adaptive immune responses. A) *Dlk1* mRNA expression in immune tissues in *Dlk1* transgenic and wild type littermate control mice measured using TaqMan PCR. Relative *Dlk1* expression was calculated in relation to the mean of housekeeping gene, beta-2 microglobulin, using 2-ΔΔCt method (N = 3–5 mice/group). B) Daily EAE scores in *Dlk1* transgenic (N = 24) and wild type littermate control (N = 20) mice after immunization with MOG demonstrate that the higher levels of *Dlk1* in transgenic mice are protective against EAE. Mean EAE clinical score (and the standard error of the mean) is given for each day post immunization (p.i.). The figure represents pooled data from three separate experiments. Mann-Whitney U-test was used to compare the daily EAE scores (p-value <0.05  =  *). C) Flow cytometry analysis of percentages of activated T helper cells (CD3+CD4+CD44+) and B cells (CD3-CD45R+) in spleen tissue 25 days after EAE induction, *ex vivo* and after 48 hour of restimulation with MOG (MOG recall). Transgenic *Dlk1* mice displayed lower percentage of activated T cells and B cells compared to wild type littermate controls (N = 7/group). D) Flow cytometry analysis of numbers of differentiated IFN-γ producing Th1 cells (details in Materials and Methods). Transgenic *Dlk1* mice showed a lower number of Th1 cells in comparison to wild type littermates (N = 2/group, three independent experiments). Green and orange histograms show IFN-γ producing cell population in control and fully Th1 differentiated conditions, respectively.

We next investigated the effect of differential *Dlk1* expression on EAE using transgenic C57BL/6 mice that express a double dosage of *Dlk1* in multiple tissues [34]. The *Dlk1* transgenic mice were created by pronuclear injection of a bacterial artificial chromosome (BAC) transgene that encompasses the entire *Dlk1* gene and endogenous flanking sequences but without the imprinting control region and the other genes in the cluster [34]. We first confirmed that levels of *Dlk1* were elevated in three different immune tissues of transgenic mice compared to wild type littermate controls (Figure 6A). As expected from the backcross data, lower expression of *Dlk1* in wild type mice predisposed to more severe EAE while higher expression of *Dlk1* in transgenic mice was protective against EAE (Figure 6B). The observed differences in clinical disease were accompanied with differences in the immune response with lower frequency of activated CD4^+^ T cells and B cells in protected transgenic mice compared to their wild type littermate controls (Figure 6C). Furthermore, during the *in vitro* differentiation of naïve T cells into IFNγ-producing Th1 cells, known to have a pathogenic role in EAE [35]–[37], we observed that transgenic mice produced lower numbers of Th1 cells compared to wild type controls (Figure 6D).

Taken together, our data demonstrate parent-of-origin effects in EAE, including imprinting-like patterns of transmission of disease-predisposing alleles. Furthermore, we show that imprinted *Dlk1* specifically modulates the adaptive immune responses and regulates susceptibility to EAE *in vivo*.

## Discussion

Our data demonstrate that a striking 37–54% of loci predisposed for EAE in a parent-of-origin dependent manner. One of the very few studies in EAE that used a reciprocal backcross design in mice similarly demonstrated that 50% of EAE loci depend on parental transmission, although this result was based on a total of two out of four identified QTLs [38]. Parent-of-origin dependent loci on chromosomes 6, 10 and 18 identified in this study have been previously reported [39]–[42] as well as the majority of identified loci that did not depend on parental transmission [22], [39]–[42]. Replication of EAE loci in independent populations and strain combinations is important as it justifies investments in further candidate gene identification, which can also be significantly facilitated by exploiting information about locus segregation between multiple inbred strains. Additionally, replication of the majority of the loci that did not depend on parental origin confirms that our study was adequately powered to investigate parent-of-origin effects. Indeed, taking into account parent-of-origin enabled identification of multiple new risk loci on chromosomes 3, 4, 5, 7, 10 and 14, which have not been previously identified in rat EAE. Also, the loci on chromosomes 4 and 5 previously displayed linkage to immunological sub-phenotypes: IgG levels and the number of MHC class II positive cells in rat CNS, respectively [40], [43], but did not link to the clinical disease phenotypes. This likely reflects the lack of power in previous studies to identify disease QTLs at these loci in populations that are confounded by the parent-of-origin.

Parent-of-origin dependent EAE loci identified in this study overlap with experimentally confirmed or clusters of highly predicted imprinted genes (Figure S2). One example is the well-studied GNAS complex locus located at the peak of the maternally transmitted EAE QTL on chromosome 3. This locus comprises multiple gene products including maternally expressed G-protein a-subunit transcripts [44] that couple many receptors to cAMP signaling that is important in the immune and the nervous systems. Other known imprinted gene clusters are contained within the maternally transmitted EAE QTL on chromosome 1, including maternally inherited insulin growth factor 2 receptor (*Igf2r*) [45]. IGF2R has been shown to have an important role in T cell activation [46]. The maternally transmitted EAE locus on chromosome 14 comprises growth factor independence 1 (*Gfi1*) predicted though not shown to be maternally expressed in mice [47]. GFI1 has recently emerged as an important transcriptional repressor involved in lymphocyte development and activation (reviewed by [48]). Further functional studies in cell types relevant for EAE pathogenesis will demonstrate if known or novel imprinted genes are EAE loci.

In this study, we addressed the locus on chromosome 6 that overlaps well known imprinted *Dlk1-Dio3* cluster. Taking parent-of-origin of inherited alleles into consideration enabled us to identify *Dlk1* as a novel candidate risk gene for EAE. Indeed, predisposition to develop more severe EAE when the risk allele was exclusively paternally transmitted strongly implicated paternally expressed genes, *Dlk1*, *Rtl1* or *Dio3*, encoded in the EAE QTL on chromosome 6 [30]. Furthermore, the paternally inherited risk allele at chromosome 6 predisposed for lower expression of *Dlk1* in spleen in both DABC and PVGBC populations and in reciprocal hybrids between DA and PVG strains. The Dlk1 protein is shown to be involved in signaling pathways like the ERK/MAPK pathway [49], [50] and the FGF signaling pathway [51]. Moreover, Dlk1 protein, which is very similar to the signaling molecules of the Notch delta family [52], is an atypical Notch ligand suggested to inhibit Notch signaling [52]–[54]. The fact that Notch signaling has been strongly implicated in EAE and MS pathogenesis [55] thus suggests that lower levels of *Dlk1* might fail to appropriately control Notch signaling thereby predisposing for a more severe disease in rats.

To directly establish a role of the *Dlk1* gene in EAE pathogenesis, we used *Dlk1* transgenic mice [34] and we demonstrated that the mice express elevated levels of *Dlk1* in several immune tissues and, importantly, develop less severe EAE. This observation may, at least in part, be attributed to a role of Dlk1 in blocking Notch signaling. Accordingly, inhibiting Notch signaling has been shown to prevent and ameliorate EAE and decrease production of the proinflammatory cytokine IFN-γ [56], [57]. Nevertheless, besides its role in the immune system, Dlk1 might affect cells in the target organ in EAE/MS as Notch has been shown to suppress oligodendrocyte differentiation [55] and Dlk1 has been shown to affect neurogenesis [58]. Thus, the exact molecular mechanisms underlying a role for *Dlk1* in EAE pathogenesis remain to be investigated. In the present study, higher *Dlk1* expression ameliorated EAE and was associated with reduced frequency of activated CD4+ T cells in peripheral lymphoid tissues. This is of particular interest since CD4+ T cells have been ascribed a driving role in EAE, which can be induced with the transfer of CD4+ T cells reactive against CNS antigens [59]. In this regard, it has been previously shown that enhanced Notch signaling increases T cell proliferation [60] and protects activated T cells from going into apoptosis [61]. Accordingly, we observed that *Dlk1* transgenic mice fail to differentiate the same number of IFN-γ secreting Th1 cells compared to the wild type controls. This can explain lower severity of EAE in *Dlk1* mice considering the well documented pathogenic role of Th1 cells [35]–[37]. Because of the known association of Notch protein with nuclear-factor-kB (NF-kB), we hypothesize that less Notch protein would be able to bind to NF-kB in the *Dlk1* transgenic mice and thereby lead to less T cell activation, in general, and less IFN-γ producing Th1 cells, in particular. Moreover, *Dlk1* transgenic mice displayed lower frequency of B cells in peripheral lymphoid tissues, which is in line with a previously demonstrated effect of *Dlk1* deletion on B cell differentiation and function [31]. In addition, Notch signaling has been shown to regulate B cell activation and differentiation into antibody secreting cells [62], [63]. This is important given that B cells exert important roles not only as antigen presenting cells that activate T cells, but also as cells that produce anti-MOG antibodies, which can cause demyelination in MOG-EAE. Our study highlights for the first time *Dlk1* as a regulator of the adaptive immune responses and an autoimmune disease that models MS.

Our findings also have important implications for genetic studies of complex human diseases, currently dominated by genome-wide association studies (GWAS) that do not take into account parental origin of alleles. It is generally accepted that GWAS have a ‘missing heritability’ component, some of which may reside in parent-of-origin effects. Our study supports that parent-of-origin should be accounted for and could be one of many explanations for why all the identified risk variants together explain typically less than 30% of heritability [64]. For example, 180 loci identified in GWAS explain around 12% of height heritability [65]. In Crohn's disease, 71 risk genes explain less than 25% of heritability [66]. Similarly, in MS we found that 61 genetic variants explain ∼20% of genetic risk for disease [67]. Kong *et al* used the detailed genealogical information and long-range phasing of haplotypes to determine the parent-of-origin of alleles in Icelanders to identify five additional SNPs in imprinted genes that associate with disease [68]. Thus, information on the parental transmission of risk alleles is likely to add to the ‘missing heritability’ of complex diseases. Our data advocate family studies that can address the impact of parent-of-origin combined with the development of more powerful statistical methods to detect parent-of-origin effects in human populations. Indeed, Wallace *et al* identified a SNP neighboring the *Dlk1* locus that strongly associates with type 1 diabetes depending on the parental origin [69] supporting our findings of a role of *Dlk1* in autoimmunity.

Taken together, these results reinforce the importance of parent-of-origin effects and demonstrate that incorporating these effects into models of inheritance not only enables more powerful and precise identification of risk factors but also can provide a better understanding of the pathogenesis of complex diseases.

## Materials and Methods

### Ethics statement

All experiments in this study were approved and performed in accordance with the guidelines from the Swedish National Board for Laboratory Animals and the European Community Council Directive (86/609/EEC) under the ethical permits N332/06, N338/09 and N298/11 entitled ‘Genetic regulation, pathogenesis and therapy of EAE, an animal model for multiple sclerosis’, which were approved by the North Stockholm Animal Ethics Committee (Stockholms Norra djurförsöksetiska nämnd). Rats were tested according to a health-monitoring program at the National Veterinary Institute (Statens Veterinärmedicinska Anstalt, SVA) in Uppsala, Sweden.

### Animals and experimental crosses

Inbred DA and PVG.AV1 rats were originally obtained from the Zentralinstitut für Versuchstierzucht (Hannover, Germany) from which colonies have been established at Karolinska Hospital (DA/Kini and PVG.1AV1/Kini). The *Dlk1* transgenic mice were generated by and originally obtained from the Ferguson-Smith laboratory (Cambridge, UK). All animals were bred and kept in 12 h light/dark- and temperature-regulated rooms. Animals were housed in polystyrene cages containing aspen wood shavings and had free access to standard rodent chow and water. Animals were tested according to a health-monitoring program at the National Veterinary Institute.

Reciprocal backcrosses were established between EAE-susceptible DA and MHC-identical EAE-resistant PVG.1AV1 strains (Figure 1). To create the F1 generation, four breeding pairs with DA female founders were established. The reciprocal N2 generation was created from DA (N = 4) and PVG.1AV1 (N = 4) females bred to F1 males and F1 females bred to DA (N = 4) or PVG.1AV1 (N = 4) males. Four N2 litters were produced for MOG-EAE experiments. The population with the susceptible DA strain (DABC) consisted of 421 rats (213 females and 208 males) and the population with the resistant PVG strain (PVGBC) consisted of 471 rats (239 females and 232 males) (Table S2).

Advanced intercross line was established from two DA and two PVG females that were bred with PVG and DA males, respectively, to produce the F1 generation. Seven F1 couples from DA female founders and seven from PVG.AV1 female founders produced the F2 generation. The G3 generation originated from 50 breeding couples and random breeding of 50 males and 50 females, avoiding brother-sister mating, produced all subsequent generations, according to Darvasi and Soller [70]. In the G10 generation, three litters similar in size were produced for MOG-EAE experiments, comprising 428 females and 366 males.

Generation and characterization of the *Dlk1* transgenic mice has previously been described in detail by da Rocha *et al.* [34]. In short, the *Dlk1* transgenic mice were created by pronuclear injection of a bacterial artificial chromosome (BAC) transgene that encompasses the entire *Dlk1* gene and endogenous flanking sequences but without other genes in the cluster. The foreign DNA is stably and randomly integrated into the genome with an estimated copy number of 4–5. Three different lines with *Dlk1* BAC transgene (70A, 70B, 70C) demonstrated no difference in phenotype indicating no impact of integration site. Transgenic *Dlk1* lines were then bred to C57BL/6 mice, known to be susceptible to EAE and extensively used as a background strain for different genetic models in EAE [71], for more than 10 generations. All experimental animals and littermate controls were derived from heterozygous *Dlk1* transgenic breeding.

### Induction and clinical evaluation of EAE

Recombinant rat and mouse MOG (amino acids 1-125 from the N terminus) was expressed in *E. Coli* and purified to homogeneity by chelate chromatography [72]. Animals were anesthetized with isoflurane (Abbott Laboratories) and immunized subcutaneously (s.c.) in the dorsal tail base. Each rat received a 200 μl inoculum containing MOG in phosphate buffered-saline (PBS) (Life Technologies) emulsified 1∶1 with incomplete Freunds adjuvant (IFA) (Sigma-Aldrich). With the aim of 50% disease incidence in each population, to achieve the highest power to detect EAE QTLs, different induction doses were used for the two backcrosses and for females and males within each backcross (Table S2). EAE was induced in 8–12 weeks old *Dlk1* transgenic and wild type littermate C57BL/6 females with 50 μg rMOG that was emulsified in Complete Freud's Adjuvant (Sigma-Aldrich) and injected s.c. in the dorsal tail base. On day zero and day two post immunization (p.i.) each mouse was injected intraperitoneally (i.p.) with Bordetella pertussis toxin (Sigma-Aldrich). Animals were weighed and clinical signs of EAE were evaluated daily from day 7 p.i. until end of experiment.

The scale for EAE scoring was: 0, healthy; 1, tail weakness or tail paralysis; 2, hind leg paresis or hemiparesis; 3, hind leg paralysis or hemiparalysis; 4, tetraplegy and 5, death. The following clinical parameters were assessed and used in analysis: incidence of EAE (INC) i.e., scored as 1 if signs of EAE were present for more than one day; onset of EAE (ONS) i.e., day of first clinical sign; duration of EAE (DUR) i.e., number of days animals showed clinical signs; maximum EAE score (MAX) and weight loss (WL), calculated by subtracting the lowest weight during the experiment from the weight at the time of immunization and expressing the difference as a percentage of the weight at the time of immunization.

### Genotyping

Genomic DNA was extracted from tail tips. Genotypes were determined by PCR amplification of microsatellite markers. Fluorophore-conjugated primers were used (Applied Biosystems, Eurofins MWG Operon) and PCR products were size fractionated on an electrophoresis capillary sequencer (ABI3730, Applied Biosystems). Genotypes were analyzed using the GeneMapper software (v. 3.7, Applied Biosystems) and all genotypes were manually confirmed by two independent observers. *Dlk1* transgenic mice were genotyped using the following primers:

*Dlk1*_wt/*Dlk1*_tg_fwd_ CCA AAC TGC ACA ACG TGC TG; *Dlk1*_wt_rev_GAT CTT GAA CTA CCA AGG GC; *Dlk1*_tg_ rev_ACT TTA TGC TTC CCG CTC GT.

### Experimental design to identify parent-of-origin effects

Two experimental populations were created by backcrossing F1 hybrids with either the susceptible DA strain (DABC) or the resistant PVG strain (PVGBC) (Figure 1). Within each population, two reciprocal crosses were established. The DAxF1breeding and the F1xDA breeding within DABC refer to the two reciprocal backcrosses. Likewise, the PVGxF1 breeding and the F1xPVG breeding within PVGBC refer to the two reciprocal backcrosses. The term “cross” always refers to one of these four reciprocal breedings, in which the first and the second strain refer to mother and father, respectively.

To identify parent-of-origin QTLs, QTL mapping was performed in the two reciprocal crosses (within the DABC or the PVGBC) separately. For example, the DAxF1 offspring inherited the PVG allele exclusively from fathers. Therefore, a QTL identified in the DAxF1 offspring and not in the F1xDA offspring would be dependent on the PVG allele predisposing for EAE only when paternally inherited. Moreover, DABC and PVGBC were used to validate parent-of-origin dependent QTLs found in each population respectively.

To control for genetic parent-of-origin effects, sex chromosomes and mitochondria varied only in DABC or PVGBC. For example, all DABC rats (offspring of the DAxF1 and the F1xDA breeding) had the DA mitochondria, while mitochondria varied between the two reciprocal crossed in the PVGBC, with PVG mitochondria in offspring from the PVGxF1 breeding and the DA mitochondria in offspring from the F1xPVG breeding. Similarly, while the Y chromosome varied between the two reciprocal crosses in DABC, all PVGBC (offspring of both the PVGxF1 and the F1xPVG breeding) were bred to have the same PVG Y chromosome.

### Statistical and linkage analysis

The genetic map was defined using publicly available genome sequence (http://www.ensembl.org/v.55). The physical map was used to enable comparison of linkage analyses between crosses and sub-populations. All animals were genotyped with 140 evenly-spaced microsatellite markers providing 97% and 91% genome coverage with 25 cM and 20 cM spacing, respectively. Linkage analysis was performed using R/qtl software [25]. A single-QTL model analysis was performed using Haley-Knott regression on phenotypes transformed to account for experimental sets [73] (data not shown). Similar results were obtained using non-transformed phenotypes as well as using non-transformed phenotypes corrected for sex, experimental set and litter size that were used as additive and interactive covariates (data not shown). Permutation tests (N = 1000) were performed to determine the threshold levels for significant linkage and genome-wide p<0.05 thresholds were reported [74]. All analyzed sub-populations had similar size (Table S2) and displayed no difference in phenotypic variation between the compared sub-populations (p>0.4 for the majority of phenotypes), apart from the weight loss in DABC males (p<0.01). Differences between phenotypic variance in the compared sub-populations were tested with two variance - F test and Levene's test in Rcmdr. In addition, all analyzed crosses had similar and sufficient power to detect QTLs (Table S3).

Due to the polygenic nature of EAE [75] we used a multiple-QTL model mapping, i.e. forward selection to a model of 10 additive QTLs followed by backward elimination to the null model to identify a multiple-QTL model. A threshold LOD for a model of choice was set to allow detection of QTLs with modest effects, which we previously identified and confirmed in the same disease and the same strain combination. The fit to a multiple-QTL model was used to statistically validate the independent effect of each identified QTL and percentage of phenotypic variance explained by identified multiple-QTL models. Similar results were obtained in populations combining both reciprocal crosses and using cross as an interactive covariate with Haley-Knott regression (data not shown). Allelic effects of QTLs identified in the multiple-QTL model were calculated in Rcmdr using Student's t-test (Table 2, Table 3) and confirmed with the non-parametric Mann-Whitney test (data not shown) for all phenotypes except for incidence that was tested with the Fisher's exact test.

To confirm the parent-of-origin dependent QTLs in DABC or PVGBC, a cross-by-QTL interaction analyses were performed. For each detected parent-of-origin QTL the fit-multiple QTL modeling was performed that allows the statistical validation of the independent effect of each identified QTL and its interactions. It does so by subtracting the effect of each QTL or QTL interaction and comparing that model to the initial model of phenotypic variance where all QTLs have a full effect. Here, we built a full model that comprised all identified QTLs (from the forward selection - backward elimination analysis, as in Table S1) and CROSS x QTL interaction terms for the QTLs that displayed parent-of-origin effect (i.e. QTLs that could be mapped only in one of the reciprocal crosses). The full model: Phenotype ∼ pQTL1 + QTL2 + pQTL3 + … + QTLn + pQTL1*CROSS + pQTL3*CROSS + CROSS (pQTL indicates QTLs that could be identified only in one of the crosses, see table and figure legends for models used). In the next stage the effect of each QTL or QTL*CROSS interaction was subtracted from the full model and the contribution of the subtracted term to the full model was evaluated and expressed in p-values. All independent QTLs showed significant contribution and were not included in Table 4. The table contains p-value of the full model and the p-value of the contribution of the each tested parent-of-origin dependent CROSS x QTL interaction. Genotypes at the estimated QTL locations were simulated by the imputation method (N = 128 simulations on the step = 1Mb) implemented in the R/qtl statistical software [25]. Similar results were obtained using linear regression (data not shown).

To confirm the parent-of-origin dependent QTLs in the G10, fit-multiple QTL modeling tested all parent-of-origin dependent QTLs from the backcross analysis that displayed linkage in the G10 and their parent-of-origin interactions. The full model: Phenotype ∼ 1:25 + 3:161 + 4:144 + 5:157 + 7:50 + 10:23 + 10:82 + 14:5 + 1:25*Origin + 3:161*Origin + 4:144 *Origin + 5:157*Origin + 7:50*Origin + 10:23*Origin + 10:82*Origin + 14:5*Origin, where first and second number refer to chromosome and peak location in Mb of parent-of-origin dependent QTLs from the backcross analysis, respectively, and “Origin” refers to G9 parental/family origin of G10 rats. Table 5 contains the p-value of the contribution of the each tested parent-of-origin dependent Origin x QTL interaction. Genotypes at the estimated QTL locations were simulated by the imputation method (N = 128 simulations on the step = 1Mb) implemented in the R/qtl statistical software [25].

To identify the most likely location of the gene of interest in the chromosome 6 QTL, we calculated the probability of the gene being located at each position using a bootstrap approach in R/qtl [25]. The imputation method was chosen as it could be used with multivariate and non-normally distributed phenotypes, covariates, missing genotype data and genotyping errors in inbred line crosses. Simulated pedigrees were sampled with replacement from the observed DABC and PVGBC individuals to create a new data set with the same number of samples (Table 1), which was mapped using a single-QTL model in R/qtl [25]. The maximum LOD and the location of that maximum were recorded and the resampling was repeated 1000 times to obtain an estimate of the probability of the QTL effect being present at each position within the confidence interval. This procedure was repeated for each phenotype.

### Sequencing

Exons of *IGF2* gene were sequenced from the genomic DNA. Primers were designed using the Oligo 6.0 software (National Biosciences). PCR was performed using Platinum Taq protocol (Invitrogen), amplified DNA was purified (Qiagen Gmbh) and sent for sequencing (Eurofins MWG Operon). Sequence alignment was performed with Vector NTI software (InforMax). The identified SNP in the 5′ UTR of *IGF2* [GeneBank:184956655] was confirmed by re-sequencing.

### Allele-specific quantitative real-time PCR

A common reverse and two allele-specific forward primers, one that ends with C, complementary to the DA *IGF2* allele and one that ends with T, complementary to the PVG *IGF2* allele, were designed using Primer Express software (Applied Biosystems). The primer sequences for *IGF2* are: forward primer, 5′ TCC TCT TGA GCA GGG ACA GC 3′ (DA allele); 5′ TCC TCT TGA GCA GGG ACA GT 3′ (PVG allele); reverse primer, 5′ AAA CCT GGG AAG GGA AGT GG 3′. The primer sequences for *HPRT* are: forward primer, 5′ CTC ATG GAC TGA TTA TGG ACA 3′; reverse primer, 5′ GCA GGT CAG CAA AGA ACT TAT 3′. Snap frozen liver tissue from new born rats was disrupted using Lysing Matrix D tubes (MP Biomedicals) on a FastPrep homogenizer (MP Biomedicals) and mRNA was extracted using RNeasy mini columns (Qiagen Gmbh), including on column DNA-digestion. Reverse transcription was performed with random hexamer primers (Gibco BRL) and Superscript Reverse Transcriptase (Invitrogen). Real-time PCR was performed on a BioRad iQ5 iCycler Detection System (BioRad) with a three-step PCR protocol (95°C for 3 min. followed by 40 cycles of 95°C for 10 sec., 67°C for 30 sec. and 72°C for 30 sec.) and with SYBR green fluorophore. PCR conditions (in specific, the annealing temperature) were optimized using DA and PVG samples to assure allele-specific amplification. At the annealing temperature of 67 degrees and using specific forward primer there was no amplification of the non-complementary allele (Ct >36). Relative quantification of mRNA levels was performed using the standard curve method, with amplification of target mRNA and HPRT mRNA. The standard curves were created using five serial 10-fold dilutions. The relative amount of mRNA in each sample was calculated as the ratio between the target mRNA and the corresponding endogenous control HPRT mRNA.

### Quantitative real-time PCR

RNA was extracted from rat and mouse tissues using Qiagen RNeasy Mini Kit and cDNA created with BioRad iScript Kit. Quantitative real-time PCR of rat *Dlk1*, *Rtl1* and *Dio3* in the BC material was performed using a BioRad CFX384 Touch real-time PCR system with a two-step PCR protocol (95°C for 3 min. followed by 40 cycles of 95°C for 10 sec., 60°C for 30 sec. followed by melt curve analysis), using SYBR Green as the fluorophore (Bio-Rad). Cycle of threshold (Ct), efficiencies and melt curves were analyzed in CFX Manager software (Bio-Rad) and relative expression was calculated in relation to the mean of housekeeping genes, hypoxanthine phosphoribosyltransferase (*Hprt*) using 2-ΔΔCt. The following primers were used: *Hprt*_fwd CTC ATG GAC TGA TTA TGG ACA, *Hprt*_rev GCA GGT CAG CAA AGA ACT TAT; *Dlk1*_fwd CGG GAA ATT CTG CGA AAT AGA T, *Dlk1*_rev TCT CGA GGT CCA CGC AAG TC; *Rtl1*_fwd GCA TCG CAC TCG AGA ACT ACA G, *Rtl1*_rev CGT CGG CCA GGT CTG AGT AT; *Dio3*_fwd CAT CTG CGT ATC CGA CGA CA, *Dio3*_rev CTC ATG GGC CTG CTT GAA GA. We also used TaqMan quantitative PCR in mice and rat F1 reciprocals to measure *Dlk1* expression and expression was normalized to *Hprt*. All qPCR reactions were carried out in 10 μl final volume using Standard Tagman qPCR conditions (Applied Biosystems protocol) and all samples were run in triplicates. *Dlk1* expression levels were measured using TaqMan gene expression assay ID Rn00587011_m1 and Mm00494477_m1 for rat and mouse *Dlk1*, respectively.

### *Ex vivo* and *in vitro* cell culture

Single cell suspension was prepared from spleen and lymph node tissue dissected 25 days p.i. and 10∧6 cells/well were plated in a 96-well V bottom plate (Corning) for FACS staining. For MOG recall 10∧6 cells/well were plated in a 96-well flat bottom plate (Corning) in RPMI medium (Life Technologies) supplemented with 10% FCS (Life technologies) and challenged with 20 μg of rMOG. After 48 h cells were transferred to a V bottom plate and prepared for FACS staining. For Th1 differentiation naïve CD4+ T cells were purified from whole lymph node cells using the CD4+ T cell isolation kit (Milteny Biotec). After isolation naïve CD4+ T cells were cultured with 1 μg/ml anti-CD3 (BD), 1 μg/ml anti-CD28 (BD) and 10 ng/ml of interleukin 12 (R&D systems) in RPMI medium supplemented with 10% FCS with and without 10 ng/ml of interleukin 2 for three days.

### Flow cytometry

To characterize different immune cell subsets cells were stained with the following markers: FITC and A700 labeled CD3, FITC and APC labeled CD4, PE and PECy7 labeled CD8, A700 labeled CD44 Texas Red labeled CD45R and V450 labeled Ki67 and FoxP3 (from BD and eBioscience). For detection of IFNγ producing Th1 cells, naïve CD4+ T cells were incubated for 4 h with PMA and Ionomycin and then stained with IFNγ APC (BD). Surface stainings were done in PBS containing LIVE/DEAD fixable far-red dead cells exclusion dye (Life Technologies) and intracellular/cytokine stainings were done with the FoxP3 staining Kit (eBioscience). Cells were acquired in a Gallios flow cytometer and analyzed with the Kaluza software (both from Beckman Coulter).

## Supporting Information

Figure S1 Parent-of-origin dependent locus that comprises the imprinted *IGF2* gene regulates weight. A) A logarithm of the odds (LOD) plot for weight at the time of immunization in DABC shows a QTL on rat chromosome 1 that regulated weight in females and males. Inserts of the allelic effect plots at the peak marker at 235 Mb (indicated by *) show that the PVG allele predisposed for higher weight in females and males. B) LOD plot of chromosome 1 in DABC rats shows an additional parent-of-origin dependent QTL at 185 Mb (indicated by *) that could be identified in DAxF1 but not in F1xDA females. C) Allelic effect plots at the peak marker at 185 Mb (indicated by * in B) show that the PVG allele predisposed for higher weight significantly more when paternally transmitted (DAxF1) compared to the DA allele. D) Weight in grams of newborn hybrid (F1) pups was higher when the PVG allele was paternally transmitted (p<0.05). E) Allele-specific expression of *IGF2* in liver tissue of newborn hybrid rats demonstrated imprinting, where the paternal copy was expressed while the maternal copy was silenced. LOD scores were generated using Haley-Knott regression (thresholds for significant linkage were 2.7, 2.6, 2.8, 2.9 and 2.8 for combined, females, males, DAxF1 females (N = 105) and PVGxF1 females (N = 119), respectively). P-values given in allelic effect plots were calculated using Student's t-test. DA and PVG alleles are indicated with D and P, respectively, and the maternally and paternally inherited alleles are indicted with m and p, respectively.
(EPS)

Figure S2 Overlap of identified parent-of-origin dependent QTLs with known and predicted imprinted genes. Histogram plots showing the frequency of confirmed (red) and predicted (grey) rat orthologs of imprinted genes (Y-axis) along with their genomic locations given in mega bases across rat chromosomes (X-axis). Black horizontal bars indicate the parent-of-origin EAE loci intervals. Identification of confirmed and predicted imprinted genes is based on the Otago database of imprinted genes http://igc.otago.ac.nz/home.html and 26 genome-wide studies (Text S2).
(EPS)

Table S1 Linkage analysis shows the polygenic nature of EAE. Linkage analysis using forward selection with reverse elimination identified QTLs on the following locations (in Mb): 1a(25), 1b(248), 3(161), 4a(144), 4b(185), 5a(25), 5b(157), 6(131), 7a(21), 7b(50), 10a(23), 10b(50), 10c(82), 10d(98), 11(47), 12(25), 13(58), 14(5), 15(82), 18(80) and 19(50). Abbreviations: N  =  number of QTLs, Chr  =  chromosome locations, Var  =  percent of phenotypic variance explained by the statistical model (Table S6), INC  =  incidence of EAE, MAX  =  maximum EAE score, DUR  =  duration of EAE, ONS  =  onset of EAE, WL  =  weight loss.
(DOC)

Table S2 Summary of experimental sets.
(DOC)

Table S3 QTL detection power for the reciprocal backcross populations. The power (%) to detect a QTL over the range of effects typical for EAE QTLs was calculated in R/qtl using 5000 simulations for several population sizes (110–130 individuals corresponds to reciprocal backcrosses in females and males separately, whereas 225–250 individuals corresponds to reciprocal backcrosses when females and males were analyzed together)(Table S2, manuscript). All parent-of-origin dependent QTLs could be detected also when analysis was done in females and males together with sex-adjusted phenotypic values (Table S1, manuscript). Bold text indicate common effect and population size for various QTLs. Similar results were obtained using power calculations in qtlDesign software.
(DOC)

Table S4 The statistical test of parent-of-origin effect in QTLs that do not show evidence of parent-of-origin. Analysis was performed using the fit-multiple QTL model. A full model comprised nine QTLs that do not show parent-of-origin effect and parent-of-origin (G9) x QTL interactions (Phenotype ∼ *Eae30* + Eae30*ORIGIN + *Eae31* + Eae31*ORIGIN + *Eae24* + Eae24*ORIGIN + *Eae26* + Eae26*ORIGIN + *Eae18b* + Eae18b*ORIGIN + *Eae5* + Eae5*ORIGIN + *Eae17* + Eae17*ORIGIN + *Eae23a* + Eae23a*ORIGIN + *Eae23b* + Eae23b*ORIGIN + ε). In the next stage the effect of each QTL or origin x QTL interaction was subtracted from the full model and the contribution of the subtracted term to the full model was evaluated and expressed in p-values. Presented in the table are only p-values for the parent-of-origin (G9) x QTL terms. Analysis was performed in 794 G10 rats. n/a, no significant evidence for a QTL.
(DOC)

Table S5 The statistical test of parent-of-origin effect in 10 random non-EAE loci. Analysis was performed using the fit-multiple QTL model. A full model comprised 10 random loci that do not show evidence for EAE in G10 or parent-of-origin in the backcross and parent-of-origin (G9) x loci interactions. The model tested was Phenotype ∼1:160+1:160*ORIGIN + 3:95 + 3:95*ORIGIN + 5:125 + 5:125*ORIGIN + 8:99 + 8:99*ORIGIN + 9:36 + 9:36*ORIGIN + 10:105 + 10:105*ORIGIN + 11:47 + 11:47*ORIGIN + 13:34 + 13:34*ORIGIN + 15:75 + 15:75*ORIGIN + 17:16 + 17:16*ORIGIN +ε, with the number indicating chromosome:location in Mb of the loci tested. In the next stage the effect of each loci or origin x loci interaction was subtracted from the full model and the contribution of the subtracted term to the full model was evaluated and expressed in p-values. Analysis was performed in 794 G10 rats.
(DOC)

Table S6 Models used for variance calculations. The models used to calculate variance in Figure 2 and Table S1. The model commonly used for linkage analyses does not account for parental origin of alleles and was used to identify QTLs in the entire populations (DABC or PVGBC). The models generated by this method are specified under Reduced Model and were used to calculate variance for DABC and PVGBC. To identify parent-of-origin QTLs, we mapped the DAxF1 or PVGxF1 separately from the F1xDA or F1xPVG, and included the QTLs that could be identified in either population in the model. These are specified under Full Model and were used to calculate variance for DAxF1 together with F1xDA and PVGxF1 together with F1xPVG. The variance that could be explained under the parent-of-origin model (Full Model) compared to a reduced model indicates that parent-of-origin contributes to explaining the EAE phenotypes.
(DOC)

Text S1 Mitochondrial effects.
(DOC)

Text S2 Supplementary references.
(DOC)
