# Supplementary material for: Parent-of-Origin Effects Implicate Epigenetic Regulation of Experimental Autoimmune Encephalomyelitis and Identify Imprinted Dlk1 as a Novel Risk Gene
Source: PLoS Genet. 2014 Mar 27;10(3):e1004265. doi: 10.1371/journal.pgen.1004265 (PMC3967983; doi:10.1371/journal.pgen.1004265)
Supplement: Table S1 — Linkage analysis shows the polygenic nature of EAE. Linkage analysis using forward selection with reverse elimination identified QTLs on the following locations (in Mb): 1a(25), 1b(248), 3(161), 4a(144), 4b(185), 5a(25), 5b(157), 6(131), 7a(21), 7b(50), 10a(23), 10b(50), 10c(82), 10d(98), 11(47), 12(25), 13(58), 14(5), 15(82), 18(80) and 19(50). Abbreviations: N = number of QTLs, Chr = chromosome locations, Var = percent of phenotypic variance explained by the statistical model (Table S6), INC = incidence of EAE, MAX = maximum EAE score, DUR = duration of EAE, ONS = onset of EAE, WL = weight loss. (DOC) [file pgen.1004265.s003.doc]

Table S1. Linkage analysis shows the polygenic nature of EAE

|  | |  | **Females and Males** | | | | | **Females** | | | | | **Males** | | | | |
| --- | --- | --- | --- | --- | --- | --- | --- | --- | --- | --- | --- | --- | --- | --- | --- | --- | --- |
|  |  | | **INC** | **MAX** | **DUR** | **ONS** | **WL** | **INC** | **MAX** | **DUR** | **ONS** | **WL** | **INC** | **MAX** | **DUR** | **ONS** | **WL** |
| **N** | **ALL** | | 2 | 4 | 2 | 4 | 4 | 6 | 5 | 4 | 5 | 5 | 2 | 2 | 2 | 4 | 3 |
| **N** | **DA x F1** | | 2 | 2 | 1 | - | 1 | 1 | 1 | 2 | 1 | 3 | - | - | - | 2 | - |
| **N** | **F1 x DA** | | - | 5 | 2 | 3 | 4 | 4 | 3 | 1 | 6 | 3 | 4 | - | - | 1 | 3 |
| **Chr** | **ALL** | | 10b, 12 | 4b,5b  10b,12 | 10b  12 | 4b  10b  12,14 | 4b,5b  10b,12 | 4b,7a,  10d,12  14,15 | 4b,5b,7a  10d,12 | 6,7a  10d,12 | 4b,7a  10d,12  14 | 4b,5b  6,10d  12 | 1b,10b | 1b,10a | 1b,10b | 10b,1114,19 | 1b,10b,11 |
| **Chr** | **DA x F1** | | 10a,12 | 10a,12 | 10a | - | 12 | 12 | 12 | 10d,12 | 12 | 6,10d  12 | - | - | - | 11,14 | - |
| **Chr** | **F1 x DA** | | - | 1b,4a,4b5b,10d | 1b, 5b | 4a,10b  14 | 1b,4a  5b,7b | 4a,7a  10d,14 | 4a,5b 10d | 5b | 4a,5b,7a 7b ,10d, 14 | 4a,5b 7b | 1b,4a  10b,15 | - | - | 10b | 1b,4a11 |
| **Var** | **ALL** | | 7.2 | 11.0 | 6.7 | 10.3 | 10.0 | 28.2 | 22.5 | 17.8 | 20.4 | 21.9 | 9.1 | 9 | 8.8 | 15.1 | 16 |
| **Var** | **DA x F1** | | 10.0 | 10.8 | 6.0 | - | 6.9 | 10.2 | 10.7 | 17.5 | 8.1 | 31.2 | - | - | - | 15.3 | - |
| **Var** | **F1 x DA** | | - | 20.7 | 8.5 | 14.2 | 20.8 | 27.8 | 28.5 | 11 | 41.7 | 26.1 | 19.2 | - | - | 10.4 | 22.5 |
| **N** | **ALL** | | 3 | 5 | 2 | 4 | 1 | 1 | 1 | 1 | 2 | 3 | 1 | 1 | 1 | 1 | - |
| **N** | **PVG x F1** | | 1 | 1 | 2 | 2 | - | - | - | 1 | 1 | - | 1 | 1 | 1 | 1 | - |
| **N** | **F1 x PVG** | | 3 | 3 | 3 | 3 | 1 | 2 | 2 | 2 | 3 | 1 | 2 | 2 | 2 | 1 | 2 |
| **Chr** | **ALL** | | 4a,5a,10a | 4a,5a,6 10a,13 | 4a,10a | 4a,6,10b 13 | 10a | 10a | 10a | 10a | 4b,10a | 10b,11 | 4a | 4a | 4a | 4a | - |
| **Chr** | **PVG x F1** | | 6 | 6 | 6,10b | 6,10c | - | - | - | 10c | 10c | - | 6 | 6 | 6 | 6 | - |
| **Chr** | **F1 x PVG** | | 1a,4a 10a | 4a,10a  18 | 1a,4a 10a | 3,4a,10b | 10a | 3,10a | 3,10a | 4a,10a | 3,4a,10a | 10b | 1a,18 | 1a,18 | 4a,18 | 4a | 1a,18 |
| **Var** | **ALL** | | 7 | 9.2 | 7.5 | 11.5 | 1.4 | 3.8 | 3.3 | 4.9 | 6.8 | 11.1 | 4.6 | 3.6 | 6.6 | 6.3 | - |
| **Var** | **PVG x F1** | | 4.0 | 4.3 | 8.0 | 8.8 | - | - | - | 7.8 | 7.6 | - | 8.7 | 7.2 | 8.6 | 9.9 | - |
| **Var** | **F1 x PVG** | | 15.0 | 13.6 | 15.5 | 13.6 | 3.4 | 14.3 | 14 | 14.9 | 20.1 | 7.4 | 16.1 | 15.0 | 14.8 | 10.4 | 14.0 |

Linkage analysis using forward selection with reverse elimination identified QTLs on the following locations (in Mb): 1a(25), 1b(248), 3(161), 4a(144), 4b(185), 5a(25), 5b(157), 6(131), 7a(21), 7b(50), 10a(23), 10b(50), 10c(82), 10d(98), 11(47), 12(25), 13(58), 14(5), 15(82), 18(80) and 19(50). Abbreviations: N = number of QTLs, Chr = chromosome locations, Var = percent of phenotypic variance explained by the statistical model (Table S6), INC = incidence of EAE, MAX = maximum EAE score, DUR = duration of EAE, ONS = onset of EAE, WL = weight loss.
