# Supplementary material for: Parent-of-Origin Effects Implicate Epigenetic Regulation of Experimental Autoimmune Encephalomyelitis and Identify Imprinted Dlk1 as a Novel Risk Gene
Source: PLoS Genet. 2014 Mar 27;10(3):e1004265. doi: 10.1371/journal.pgen.1004265 (PMC3967983; doi:10.1371/journal.pgen.1004265)
Supplement: Table S2 — Summary of experimental sets. (DOC) [file pgen.1004265.s004.doc]

**Table S2. Summary of experimental sets**

|  |  | **DABC** | | | | **PVGBC** | | | |
| --- | --- | --- | --- | --- | --- | --- | --- | --- | --- |
| **Set** | **Sex** | **MOG**  **(µg/rat)** | **DA x F1** | **F1 x DA** | **N** | **MOG**  **(µg/rat)** | **PVG x F1** | **F1 x PVG** | **N** |
| **1** | Females | 12.5 | 25 | 24 | 49 | 40 | 18 | 19 | 37 |
|  | Males | 30 | 19 | 23 | 42 | 80 | 17 | 19 | 36 |
| **2** | Females | 15 | 17 | 26 | 43 | 25 | 26 | 25 | 51 |
|  | Males | 25 | 18 | 29 | 47 | 45 | 22 | 26 | 48 |
| **3** | Females | 14 | 36 | 32 | 68 | 25 | 21 | 23 | 44 |
|  | Males | 25 | 40 | 27 | 67 | 45 | 19 | 27 | 46 |
| **4** | Females | 14 | 27 | 26 | 53 | 25 | 20 | 20 | 40 |
|  | Males | 25 | 27 | 25 | 52 | 45 | 14 | 31 | 45 |
| **5** | Females | - | - | - | - | 25 | 34 | 33 | 67 |
|  | Males | - | - | - | - | 45 | 34 | 23 | 57 |
| **Σ** |  |  | 209 | 212 | 421 |  | 225 | 246 | 471 |
