# Supplementary material for: Parent-of-Origin Effects Implicate Epigenetic Regulation of Experimental Autoimmune Encephalomyelitis and Identify Imprinted Dlk1 as a Novel Risk Gene
Source: PLoS Genet. 2014 Mar 27;10(3):e1004265. doi: 10.1371/journal.pgen.1004265 (PMC3967983; doi:10.1371/journal.pgen.1004265)
Supplement: Table S3 — QTL detection power for the reciprocal backcross populations. The power (%) to detect a QTL over the range of effects typical for EAE QTLs was calculated in R/qtl using 5000 simulations for several population sizes (110–130 individuals corresponds to reciprocal backcrosses in females and males separately, whereas 225–250 individuals corresponds to reciprocal backcrosses when females and males were analyzed together)(Table S2, manuscript). All parent-of-origin dependent QTLs could be detected also when analysis was done in females and males together with sex-adjusted phenotypic values (Table S1, manuscript). Bold text indicate common effect and population size for various QTLs. Similar results were obtained using power calculations in qtlDesign software. (DOC) [file pgen.1004265.s005.doc]

**Table S3. QTL detection power for the reciprocal backcross populations**

|  | Size | Effect | 5% | 6% | 7% | 8% | 9% | 10% | 12% |
| --- | --- | --- | --- | --- | --- | --- | --- | --- | --- |
| ♀ or ♂ | 110 |  |  |  |  | 60 | 69 | **71** | **84** |
| ♀ or ♂ | 120 |  |  |  |  | 64 | **70** | **76** | 85 |
| ♀ or ♂ | 130 |  |  |  |  | 70 | **74** | **81** | 89 |
| ♀ and ♂ | 225 |  | 72 | **81** | **88** | 92 |  |  |  |
| ♀ and ♂ | 250 |  | **74** | **86** | 90 | 96 |  |  |  |

The power (%) to detect a QTL over the range of effects typical for EAE QTLs was calculated in R/qtl using 5000 simulations for several population sizes (110‐130 individuals corresponds to reciprocal backcrosses in females and males separately, whereas 225‐250 individuals corresponds to reciprocal backcrosses when females and males were analyzed together)(Table S2, manuscript). All parent‐of‐origin dependent QTLs could be detected also when analysis was done in females and males together with sex‐adjusted phenotypic values (Table S1, manuscript). Bold text indicate common effect and population size for various QTLs. Similar results were obtained using power calculations in qtlDesign software.
