# Supplementary material for: Parent-of-Origin Effects Implicate Epigenetic Regulation of Experimental Autoimmune Encephalomyelitis and Identify Imprinted Dlk1 as a Novel Risk Gene
Source: PLoS Genet. 2014 Mar 27;10(3):e1004265. doi: 10.1371/journal.pgen.1004265 (PMC3967983; doi:10.1371/journal.pgen.1004265)
Supplement: Table S4 — The statistical test of parent-of-origin effect in QTLs that do not show evidence of parent-of-origin. Analysis was performed using the fit-multiple QTL model. A full model comprised nine QTLs that do not show parent-of-origin effect and parent-of-origin (G9) x QTL interactions (Phenotype ∼ Eae30 + Eae30*ORIGIN + Eae31 + Eae31*ORIGIN + Eae24 + Eae24*ORIGIN + Eae26 + Eae26*ORIGIN + Eae18b + Eae18b*ORIGIN + Eae5 + Eae5*ORIGIN + Eae17 + Eae17*ORIGIN + Eae23a + Eae23a*ORIGIN + Eae23b + Eae23b*ORIGIN + ε). In the next stage the effect of each QTL or origin x QTL interaction was subtracted from the full model and the contribution of the subtracted term to the full model was evaluated and expressed in p-values. Presented in the table are only p-values for the parent-of-origin (G9) x QTL terms. Analysis was performed in 794 G10 rats. n/a, no significant evidence for a QTL. (DOC) [file pgen.1004265.s006.doc]

**Table S4. The statistical test of parent-of-origin effect in QTLs that do not show evidence of parent-of-origin**

| Pheno | ***Eae30***  **1:127** | ***Eae31***  **1:190** | ***Eae24***  **4:70** | ***Eae26***  **4:96** | ***Eae18b***  **10:60** | ***Eae5***  **12:23** | ***Eae17***  **13:47** | ***Eae23a***  **17:47** | ***Eae23b***  **17:59** |
| --- | --- | --- | --- | --- | --- | --- | --- | --- | --- |
| INC | 0.3 | 0.2 | 0.2 | 0.4 | 0.2 | 0.8 | 0.5 | 0.2 | 0.6 |
| MAX | 0.5 | n/a | n/a | 0.3 | 0.2 | 0.8 | 0.3 | 0.3 | 0.4 |
| DUR | 0.3 | n/a | n/a | 0.1 | 0.2 | 0.9 | 0.2 | 0.1 | 0.6 |
| ONS | 0.3 | 0.2 | 0.2 | 0.4 | 0.3 | n/a | 0.4 | 0.1 | 0.6 |
| WL | n/a | n/a | n/a | 0.8 | 0.3 | 0.8 | 0.4 | 0.2 | n/a |

Analysis was performed using the fit-multiple QTL model. A full model comprised nine QTLs that do not show parent-of-origin effect and parent-of-origin (G9) x QTL interactions (Phenotype ~ *Eae30* + *Eae30**ORIGIN + *Eae31* + *Eae31**ORIGIN + *Eae24* + *Eae24**ORIGIN + *Eae26* + *Eae26**ORIGIN + *Eae18b* + *Eae18b**ORIGIN + *Eae5* + *Eae5**ORIGIN + *Eae17* + *Eae17**ORIGIN + *Eae23a* + *Eae23a**ORIGIN + *Eae23b* + *Eae23b**ORIGIN + ε). In the next stage the effect of each QTL or origin x QTL interaction was subtracted from the full model and the contribution of the subtracted term to the full model was evaluated and expressed in p-values. Presented in the table are only p-values for the parent-of-origin (G9) x QTL terms. Analysis was performed in 794 G10 rats. n/a, no significant evidence for a QTL.
