# Supplementary material for: Parent-of-Origin Effects Implicate Epigenetic Regulation of Experimental Autoimmune Encephalomyelitis and Identify Imprinted Dlk1 as a Novel Risk Gene
Source: PLoS Genet. 2014 Mar 27;10(3):e1004265. doi: 10.1371/journal.pgen.1004265 (PMC3967983; doi:10.1371/journal.pgen.1004265)
Supplement: Table S5 — The statistical test of parent-of-origin effect in 10 random non-EAE loci. Analysis was performed using the fit-multiple QTL model. A full model comprised 10 random loci that do not show evidence for EAE in G10 or parent-of-origin in the backcross and parent-of-origin (G9) x loci interactions. The model tested was Phenotype ∼1:160+1:160*ORIGIN + 3:95 + 3:95*ORIGIN + 5:125 + 5:125*ORIGIN + 8:99 + 8:99*ORIGIN + 9:36 + 9:36*ORIGIN + 10:105 + 10:105*ORIGIN + 11:47 + 11:47*ORIGIN + 13:34 + 13:34*ORIGIN + 15:75 + 15:75*ORIGIN + 17:16 + 17:16*ORIGIN +ε, with the number indicating chromosome:location in Mb of the loci tested. In the next stage the effect of each loci or origin x loci interaction was subtracted from the full model and the contribution of the subtracted term to the full model was evaluated and expressed in p-values. Analysis was performed in 794 G10 rats. (DOC) [file pgen.1004265.s007.doc]

**Table S5. The statistical test of parent-of-origin effect in 10 random non-EAE loci**

| Pheno | **1:160** | **3:95** | **5:125** | **8:99** | **9:36** | **10:105** | **11:47** | **13:34** | **15:75** | **17:16** |
| --- | --- | --- | --- | --- | --- | --- | --- | --- | --- | --- |
| INC | 0.5 | 0.8 | 0.5 | 0.4 | 0.6 | 0.3 | 0.5 | 0.1 | 0.7 | 0.5 |
| MAX | 0.4 | 0.9 | 0.6 | 0.7 | 0.3 | 0.1 | 0.4 | **0.05** | 0.3 | 0.7 |
| DUR | 0.5 | 0.7 | 0.6 | 0.6 | 0.4 | 0.1 | 0.6 | 0.08 | 0.2 | 0.5 |
| ONS | 0.6 | 0.7 | 0.4 | 0.4 | 0.5 | 0.5 | 0.5 | 0.2 | 0.6 | 0.5 |
| WL | 0.5 | 0.8 | **0.03** | 0.4 | 0.1 | 0.4 | 0.4 | 0.3 | 0.1 | 0.3 |

Analysis was performed using the fit-multiple QTL model. A full model comprised 10 random loci that do not show evidence for EAE in G10 or parent-of-origin in the backcross and parent-of-origin (G9) x loci interactions. The model tested was Phenotype ~ 1:160 + 1:160*ORIGIN + 3:95 + 3:95*ORIGIN + 5:125 + 5:125*ORIGIN + 8:99 + 8:99*ORIGIN + 9:36 + 9:36*ORIGIN + 10:105 + 10:105*ORIGIN + 11:47 + 11:47*ORIGIN + 13:34 + 13:34*ORIGIN + 15:75 + 15:75*ORIGIN + 17:16 + 17:16*ORIGIN + ε, with the number indicating chromosome:location in Mb of the loci tested. In the next stage the effect of each loci or origin x loci interaction was subtracted from the full model and the contribution of the subtracted term to the full model was evaluated and expressed in p-values. Analysis was performed in 794 G10 rats.
