# Supplementary material for: Parent-of-Origin Effects Implicate Epigenetic Regulation of Experimental Autoimmune Encephalomyelitis and Identify Imprinted Dlk1 as a Novel Risk Gene
Source: PLoS Genet. 2014 Mar 27;10(3):e1004265. doi: 10.1371/journal.pgen.1004265 (PMC3967983; doi:10.1371/journal.pgen.1004265)
Supplement: Table S6 — Models used for variance calculations. The models used to calculate variance in Figure 2 and Table S1. The model commonly used for linkage analyses does not account for parental origin of alleles and was used to identify QTLs in the entire populations (DABC or PVGBC). The models generated by this method are specified under Reduced Model and were used to calculate variance for DABC and PVGBC. To identify parent-of-origin QTLs, we mapped the DAxF1 or PVGxF1 separately from the F1xDA or F1xPVG, and included the QTLs that could be identified in either population in the model. These are specified under Full Model and were used to calculate variance for DAxF1 together with F1xDA and PVGxF1 together with F1xPVG. The variance that could be explained under the parent-of-origin model (Full Model) compared to a reduced model indicates that parent-of-origin contributes to explaining the EAE phenotypes. (DOC) [file pgen.1004265.s008.doc]

**Table S6. Models used for variance calculations**

|  | **DABC** | | **PVGBC** | | | |
| --- | --- | --- | --- | --- | --- | --- |
| **Phenotype** | **Reduced Model** | **Full Model** | **Reduced Model** | | | **Full Model** |
| **Females** | | | | | | |
| **INC** | QTL4b_(DABC)_ + QTL7a_(DABC)_ + QTL10d_(DABC)_ + QTL12_(DABC)_ + QTL14_(DABC)_ + QTL15_(DABC)_ + ε | QTL4a_(F1xDA)_ + QTL7a_(F1xDA)_ + QTL10d_(F1xDA)_ + QTL12_(DAxF1)_ + QTL14_(F1xDA)_ + ε | | QTL10a_(PVGBC)_ + ε | QTL3_(F1xPVG)_ + QTL10a_(F1xPVG)_ + ε | |
| **MAX** | QTL4b_(DABC)_ + QTL5b_(DABC)_ + QTL7a_(DABC)_ + QTL10d_(DABC)_ + QTL12_(DABC)_ + ε | QTL4a_(F1xDA)_ + QTL5b_(F1xDA)_ + QTL10d_(F1xDA)_ + QTL12_(DAxF1)_ + ε | | QTL10a_(PVGBC)_ + ε | QTL3_(F1xPVG)_ + QTL10a_(F1xPVG)_ + ε | |
| **DUR** | QTL6_(DABC)_ + QTL7a_(DABC)_ + QTL10d_(DABC)_ + QTL12_(DABC)_ + ε | QTL5b_(F1xDA)_ + QTL10d_(DAxF1)_ + QTL12_(DAxF1)_ + ε | | QTL10a_(PVGBC)_ + ε | QTL4a_(F1xPVG)_ + QTL10a_(F1xPVG)_ + QTL10c_(PVGxF1)_ + ε | |
| **ONS** | QTL4b_(DABC)_ + QTL7a_(DABC)_ + QTL10d_(DABC)_ + QTL12_(DABC)_ + QTL14_(DABC)_ + ε | QTL4a_(F1xDA)_ + QTL5b_(F1xDA)_ + QTL7a_(F1xDA)_ + QTL7b_(F1xDA)_ + QTL10d_(F1xDA)_ + QTL12_(DAxF1)_ + QTL14_(F1xDA)_ + ε | | QTL4b_(PVGBC)_ + QTL10a_(PVGBC)_ + ε | QTL3_(F1xPVG)_ + QTL4a_(F1xPVG)_ + QTL10a_(F1xPVG)_ + QTL10c_(PVGxF1)_ + ε | |
| **WL** | QTL4b_(DABC)_ + QTL5b_(DABC)_ + QTL6_(DABC)_ + QTL10d_(DABC)_ + QTL12_(DABC)_ + ε | QTL4a_(F1xDA)_ + QTL5b_(F1xDA)_ + QTL6_(DAxF1)_ + QTL7b_(F1xDA)_ + QTL10d_(DAxF1)_+ QTL12_(DAxF1)_ + ε | | QTL10b_(PVGBC)_ + QTL11_(PVGBC)_ + ε | QTL10b_(F1xPVG)_ + ε | |
| **Males** | | | | | | |
| **INC** | QTL1b_(DABC)_ + QTL10b_(DABC)_ + ε | QTL1b_(F1xDA)_ + QTL4a_(F1xDA)_ + QTL10b_(F1xDA)_ + QTL15_(F1xDA)_ + ε | | QTL4a_(PVGBC)_ + ε | QTL1a_(F1xPVG)_ + QTL6_(PVGxF1)_ + QTL18_(F1xPVG)_ + ε | |
| **MAX** |  |  | | QTL4a_(PVGBC)_ + ε | QTL1a_(F1xPVG)_ + QTL6_(PVGxF1)_ + QTL18_(F1xPVG)_ + ε | |
| **DUR** |  |  | | QTL4a_(PVGBC)_ + ε | QTL4a_(F1xPVG)_ + QTL6_(PVGxF1)_ + QTL18_(F1xPVG)_ + ε | |
| **ONS** | QTL10b_(DABC)_ + QTL11_(DABC)_ + QTL14_(DABC)_ + QTL19_(DABC)_ + ε | QTL10b_(F1xDA)_ + QTL11_(DAxF1)_ + QTL14_(DAxF1)_ + ε | | QTL4a_(PVGBC)_ + ε | QTL4a_(F1xPVG)_ + QTL6_(PVGxF1)_ + ε | |
| **WL** | QTL1b_(DABC)_ + QTL10b_(DABC)_ + QTL11_(DABC)_ + ε | QTL1b_(F1xDA)_ + QTL4a_(F1xDA)_ + QTL11_(F1xDA)_ + ε | |  | QTL1a_(F1xPVG)_ + QTL18_(F1xPVG)_ + ε | |

The models used to calculate variance in Figure 2 and Table S1. The model commonly used for linkage analyses does not account for parental origin of alleles and was used to identify QTLs in the entire populations (DABC or PVGBC). The models generated by this method are specified under Reduced Model and were used to calculate variance for DABC and PVGBC. To identify parent-of-origin QTLs, we mapped the DAxF1 or PVGxF1 separately from the F1xDA or F1xPVG, and included the QTLs that could be identified in either population in the model. These are specified under Full Model and were used to calculate variance for DAxF1 together with F1xDA and PVGxF1 together with F1xPVG. The variance that could be explained under the parent-of-origin model (Full Model) compared to a reduced model indicates that parent-of-origin contributes to explaining the EAE phenotypes.
