# Supplementary material for: Parent-of-Origin Effects Implicate Epigenetic Regulation of Experimental Autoimmune Encephalomyelitis and Identify Imprinted Dlk1 as a Novel Risk Gene
Source: PLoS Genet. 2014 Mar 27;10(3):e1004265. doi: 10.1371/journal.pgen.1004265 (PMC3967983; doi:10.1371/journal.pgen.1004265)
Supplement: Text S1 — Mitochondrial effects. (DOC) [file pgen.1004265.s009.doc]

**Text S1. Mitochondrial Effects**

A suggestive case of mitochondrial influence was observed for a locus on chromosome 10 (peak at 23 Mb). This QTL displayed linkage to all EAE phenotypes in DAxF1, F1xDA and F1xPVG crosses, which shared the DA mitochondria, but not in the PVGxF1 cross that has PVG mitochondria (Table 2, Table 3). However, this QTL did not display significant cross-by-QTL interaction (Table 4, Table 5), although this could be attributed to a lack of resolution to separate the effects of multiple QTLs, such as the several QTLs on chromosome 10. Thus, this QTL could depend on the mitochondrial genome of the susceptible strain.

Additional parent-of-origin effects coming from the mitochondrial genome might be implicated by the observation that 54% of loci displayed parent-of-origin effect in the PVGBC in which reciprocal crosses had different mitochondria compared to 37% of such loci in the DABC in which reciprocal crosses shared the same mitochondria. However, 75% (3/4) of QTLs that displayed maternal transmission were also identified in the DABC, ruling out mitochondrial interaction with these QTLs (Table 2, Table 3).

Thus, we found weak indications that some genomic loci might depend on the mitochondrial genome to confer EAE susceptibility and severity, however the evidence was not conclusive.
