# Supplementary material for: Parent-of-Origin Effects Implicate Epigenetic Regulation of Experimental Autoimmune Encephalomyelitis and Identify Imprinted Dlk1 as a Novel Risk Gene
Source: PLoS Genet. 2014 Mar 27;10(3):e1004265. doi: 10.1371/journal.pgen.1004265 (PMC3967983; doi:10.1371/journal.pgen.1004265)
Supplement: Text S2 — Supplementary references. (DOC) [file pgen.1004265.s010.doc]

**Text S2. Supplementary References**

1. Choi JD, Underkoffler LA, Collins JN, Marchegiani SM, Terry NA, et al. (2001) Microarray expression profiling of tissues from mice with uniparental duplications of chromosomes 7 and 11 to identify imprinted genes. Mamm Genome 12: 758-764.

2. Mizuno Y, Sotomaru Y, Katsuzawa Y, Kono T, Meguro M, et al. (2002) Asb4, Ata3, and Dcn are novel imprinted genes identified by high-throughput screening using RIKEN cDNA microarray. Biochem Biophys Res Commun 290: 1499-1505.

3. Smith RJ, Dean W, Konfortova G, Kelsey G (2003) Identification of novel imprinted genes in a genome-wide screen for maternal methylation. Genome Res 13: 558-569.

4. Luedi PP, Hartemink AJ, Jirtle RL (2005) Genome-wide prediction of imprinted murine genes. Genome Res 15: 875-884.

5. Schulz R, Menheniott TR, Woodfine K, Wood AJ, Choi JD, et al. (2006) Chromosome-wide identification of novel imprinted genes using microarrays and uniparental disomies. Nucleic Acids Res 34: e88.

6. Luedi PP, Dietrich FS, Weidman JR, Bosko JM, Jirtle RL, et al. (2007) Computational and experimental identification of novel human imprinted genes. Genome Res 17: 1723-1730.

7. Pollard KS, Serre D, Wang X, Tao H, Grundberg E, et al. (2008) A genome-wide approach to identifying novel-imprinted genes. Hum Genet 122: 625-634.

8. Wang X, Sun Q, McGrath SD, Mardis ER, Soloway PD, et al. (2008) Transcriptome-wide identification of novel imprinted genes in neonatal mouse brain. PLoS One 3: e3839.

9. Kuzmin A, Han Z, Golding MC, Mann MR, Latham KE, et al. (2008) The PcG gene Sfmbt2 is paternally expressed in extraembryonic tissues. Gene Expr Patterns 8: 107-116.

10. Babak T, Deveale B, Armour C, Raymond C, Cleary MA, et al. (2008) Global survey of genomic imprinting by transcriptome sequencing. Curr Biol 18: 1735-1741.

11. Brideau CM, Eilertson KE, Hagarman JA, Bustamante CD, Soloway PD (2010) Successful computational prediction of novel imprinted genes from epigenomic features. Mol Cell Biol 30: 3357-3370.

12. Gregg C, Zhang J, Weissbourd B, Luo S, Schroth GP, et al. (2010) High-resolution analysis of parent-of-origin allelic expression in the mouse brain. Science 329: 643-648.

13. Sritanaudomchai H, Ma H, Clepper L, Gokhale S, Bogan R, et al. (2010) Discovery of a novel imprinted gene by transcriptional analysis of parthenogenetic embryonic stem cells. Hum Reprod 25: 1927-1941.

14. Choufani S, Shapiro JS, Susiarjo M, Butcher DT, Grafodatskaya D, et al. (2011) A novel approach identifies new differentially methylated regions (DMRs) associated with imprinted genes. Genome Res 21: 465-476.

15. Nakabayashi K, Trujillo AM, Tayama C, Camprubi C, Yoshida W, et al. (2011) Methylation screening of reciprocal genome-wide UPDs identifies novel human-specific imprinted genes. Hum Mol Genet 20: 3188-3197.

16. Morcos L, Ge B, Koka V, Lam KC, Pokholok DK, et al. (2011) Genome-wide assessment of imprinted expression in human cells. Genome Biol 12: R25.

17. Wang X, Soloway PD, Clark AG (2011) A survey for novel imprinted genes in the mouse placenta by mRNA-seq. Genetics 189: 109-122.

18. Barbaux S, Gascoin-Lachambre G, Buffat C, Monnier P, Mondon F, et al. (2012) A genome-wide approach reveals novel imprinted genes expressed in the human placenta. Epigenetics 7: 1079-1090.

19. Okae H, Hiura H, Nishida Y, Funayama R, Tanaka S, et al. (2012) Re-investigation and RNA sequencing-based identification of genes with placenta-specific imprinted expression. Hum Mol Genet 21: 548-558.

20. Xie W, Barr CL, Kim A, Yue F, Lee AY, et al. (2012) Base-resolution analyses of sequence and parent-of-origin dependent DNA methylation in the mouse genome. Cell 148: 816-831.

21. Stelzer Y, Ronen D, Bock C, Boyle P, Meissner A, et al. (2013) Identification of novel imprinted differentially methylated regions by global analysis of human-parthenogenetic-induced pluripotent stem cells. Stem Cell Reports 1: 79-89.

22. DeVeale B, van der Kooy D, Babak T (2012) Critical evaluation of imprinted gene expression by RNA-Seq: a new perspective. PLoS Genet 8: e1002600.

23. Lagarrigue S, Martin LJ, Hormozdiari F, Roux PF, Pan C, et al. (2013) Analysis of Allele Specific Expression in Mouse Liver by RNA-Seq: A Comparison with "cis"-eQTL Identified Using Genetic Linkage. Genetics.

24. Zhang B, Zhou Y, Lin N, Lowdon RF, Hong C, et al. (2013) Functional DNA methylation differences between tissues, cell types, and across individuals discovered using the M&M algorithm. Genome Res 23: 1522-1540.

25. Das R, Lee YK, Strogantsev R, Jin S, Lim YC, et al. (2013) DNMT1 and AIM1 Imprinting in human placenta revealed through a genome-wide screen for allele-specific DNA methylation. BMC Genomics 14: 685.

26. Prickett AR, Barkas N, McCole RB, Hughes S, Amante SM, et al. (2013) Genome-wide and parental allele-specific analysis of CTCF and cohesin DNA binding in mouse brain reveals a tissue-specific binding pattern and an association with imprinted differentially methylated regions. Genome Res 23: 1624-1635.
